# Two-step Synthesis of Solasodine Pivalate from Diosgenin Pivalate

**DOI:** 10.3390/molecules24061132

**Published:** 2019-03-21

**Authors:** Agnieszka Wojtkielewicz, Urszula Kiełczewska, Jacek W. Morzycki

**Affiliations:** Institute of Chemistry, University of Białystok, K. Ciołkowskiego 1K, 15-245 Białystok, Poland; ulakielczewska@interia.eu (U.K.); morzycki@uwb.edu.pl (J.W.M.)

**Keywords:** solasodine, tomatidinol, diosgenin, ketal cleavage, spirostanes, steroids

## Abstract

A two-step synthesis of solasodine pivalate from diosgenin pivalate is described. The key transformation involves the reaction of diosgenin pivalate with benzyl carbamate (CbzNH_2_) promoted by TMSOTf. During the reaction the F-ring of the spiroketal moiety opens up with a simultaneous introduction of a Cbz-protected amino group in position 26. A one-pot deprotection of 26-amine with AcBr/BuOH followed by the *N*-cyclization affords solasodine pivalate in 45% overall yield.

## 1. Introduction

**Figure 1:**
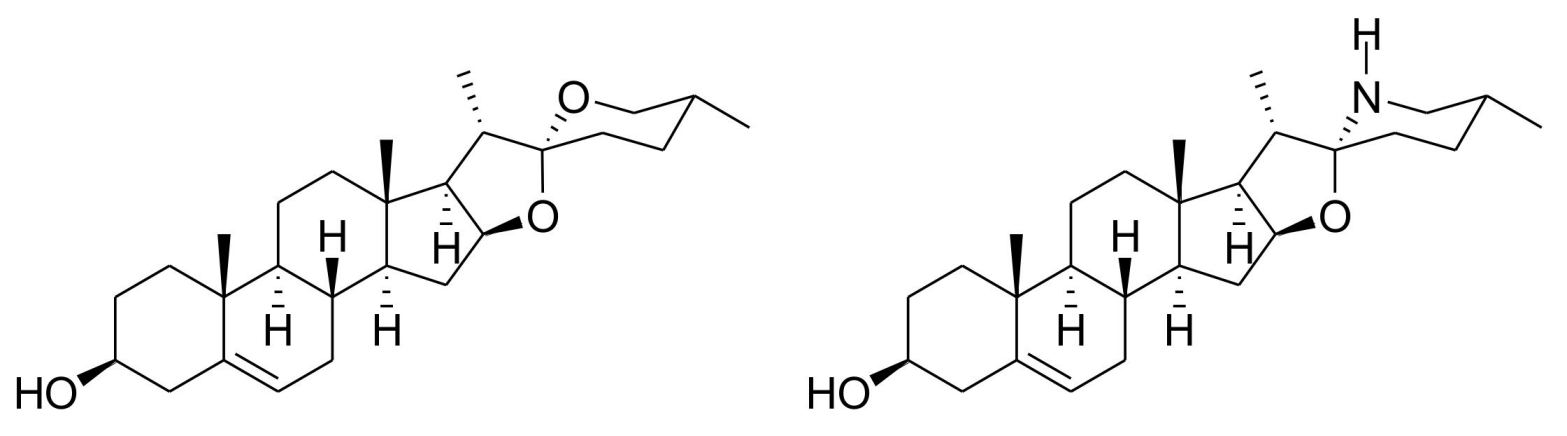
Diosgenin and solasodine structures.

Solasodine is a main representative of the *Solanum* alkaloids of spirosolane type naturally occurring in the *Solanaceae* flowering plant family (Figure 1) [1,2]. Solasodine is perceived as a pharmacologically important compound. Solasodine and its derivatives show antiproliferative [3,4,5,6], neurogenesis [7], antifungal [8], and anti-inflammatory [9] activities. Solasodine acetate is reported to significantly damage DNA and to increase DNA repair activity [10]. Coramsine, which is a mixture of solasodine glycosides, solasonine and solamargine, isolated from *Solanum linnaeanum* (devil’s apple), was tested for the treatment of melanoma [11,12]. Many of *Solanum* species containing solasodine glycosides are widely used in the traditional medicine for treatment of diverse ailments (diabetes, cholera, bronchitis, high blood pressure) and as laxatives [13].

**Scheme 1:**
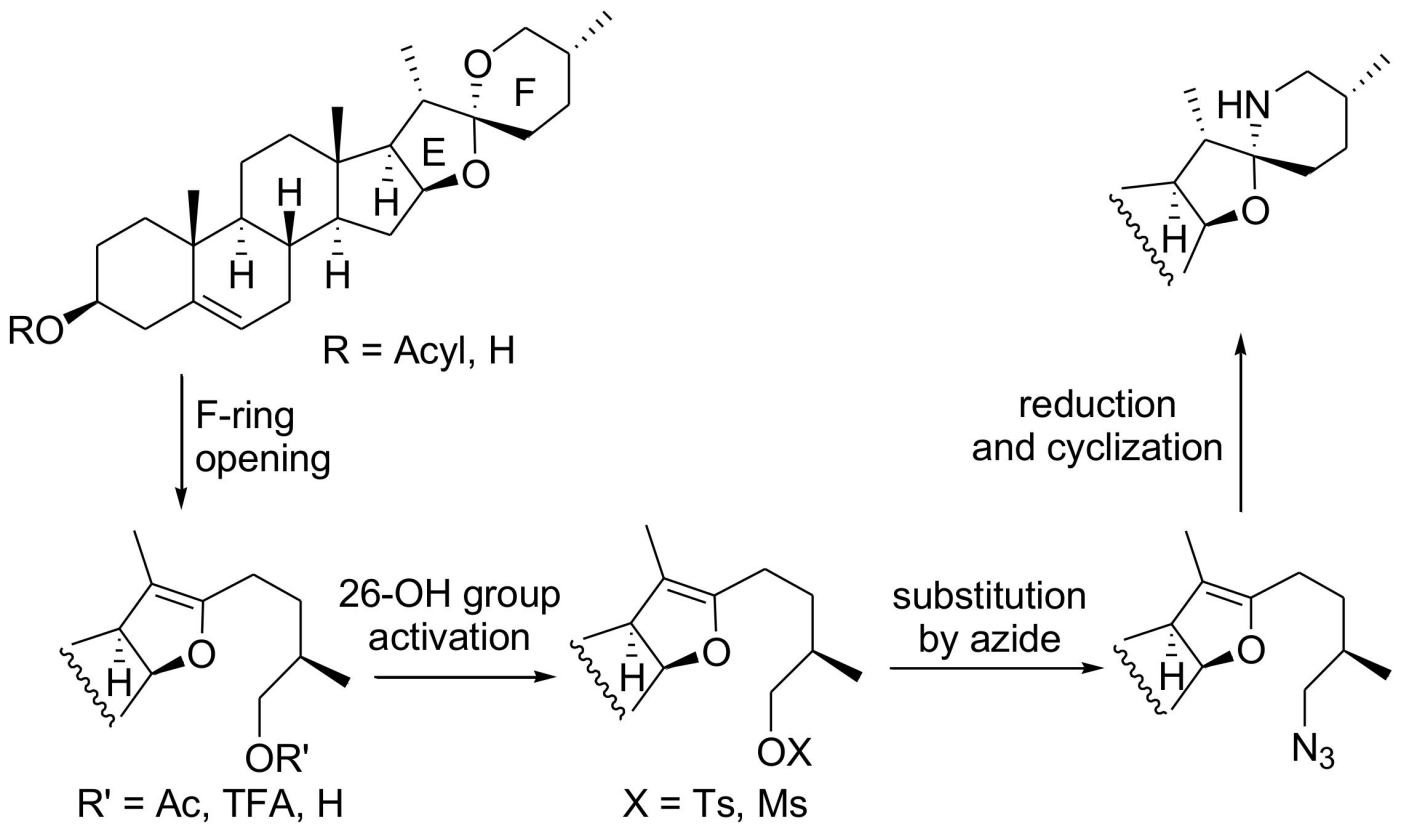
Strategy used for solasodine synthesis from diosgenin.

The significant therapeutic activities of solasodine and its derivatives and their limited availability from natural sources (solasodine usually occurs at ca. 0.03% in the various plant extracts [14]) have encouraged chemists to develop efficient methods for the synthesis of these alkaloids. Several syntheses of solasodine have been recently reported [14,15,16,17,18,19,20,21,22,23]. Most of them start from easily available diosgenin and involve F-ring opening of the steroid spiroketal system, activation of 26-OH, introduction of an *N*-containing group in position 26, its transformation into a NH_2_ moiety and finally the F-ring closure (Scheme 1). The methods employing this synthetic strategy differ mainly in reagents used for cleavage of the F-ring (e.g., Ac_2_O/AcCl [14], BF_3_xOEt_2_/Et_3_SiH [20], TFAT [21], BF_3_xOEt_2_ [22,23]). In 2015 Tian et al. reported two syntheses of solasodine from diosgenin which brought about a significant improvement in this strategy [22,23]. The first proposed method allowed shortening the solasodine preparation by bypassing the pseudodiosgenin 26-OH group activation step. Simultaneous F-ring opening and introduction of bromide in position 26 in one step led to 26-bromopseudodiosgenin, which was subsequently reacted with azide, followed by simultaneous azide reduction and *N*-cyclization of the obtained 26-amine to afford solasodine in 50% total yield [22]. Further improvement was achieved by introducing a direct C26-amination of diosgenin with sulfonamide in the presence of BF_3_xOEt_2_ [23]. Using this procedure Tian synthesized solasodine in three steps in 43% overall yield. However, the last method suffers from a few drawbacks, such as not very satisfactory yield, a troublesome removal of the sulfonyl group requiring use of metallic sodium, and the *N*-cyclization of the 26-amine as a separate step.

Keeping these shortcomings in mind, we sought a more convenient and efficient way of synthesizing solasodine. On the basis of the Tian group’s discovery [23], we decided to study diosgenin reactions with other nitrogen nucleophiles in the presence of Lewis acids. The nitrogen reagents planned for these experiments should exhibit relatively high nucleophilicity and low basicity because of the acidic conditions required for a diosgenin F-ring opening. Apart from various amides and sulfamides, azides and carbamates also show such a reactivity profile. In previous work we proved that the diosgenin reaction with TMSN_3_ under TMSOTf catalysis took an unexpected course and led to furostane-26-nitrile in a single step [24]. Herein, we report a short and efficient synthesis of solasodine, in which the key intermediate is the 26-benzyloxycarbonyl-aminopseudodiosgenin derivative obtained in the reaction of diosgenin with benzyl carbamate in the presence of TMSOTf.

## 2. Results and Discussion

**Scheme 2:**
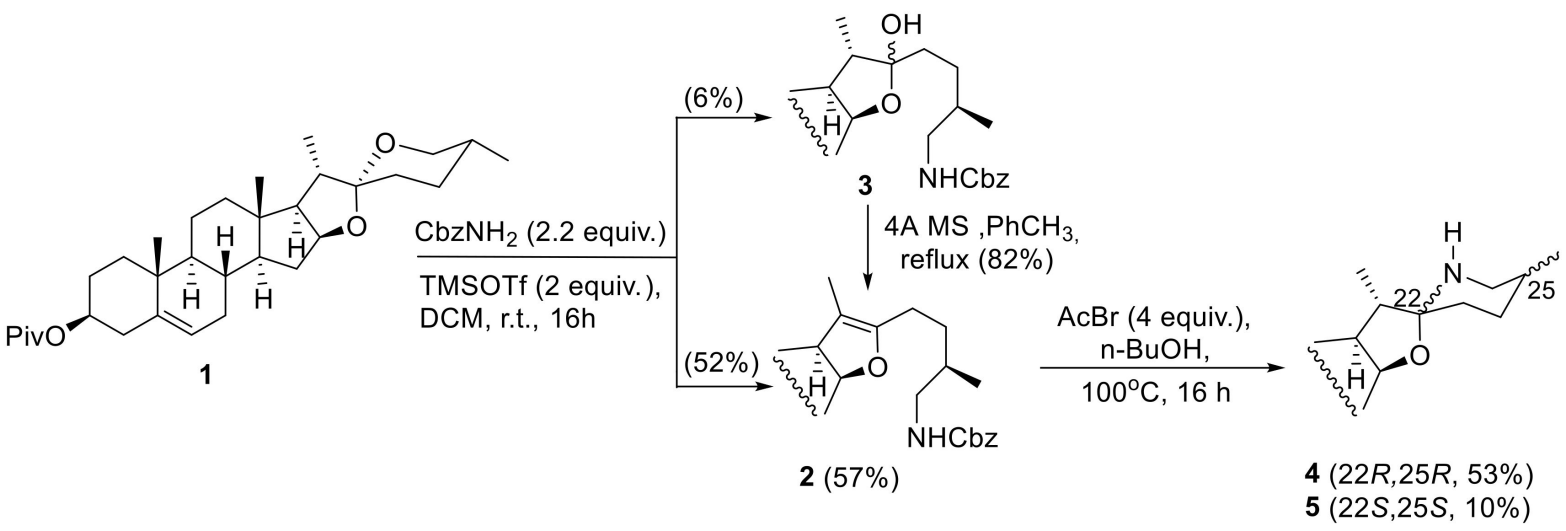
Solasodine pivalate synthesis employing diosgenin reaction with CbzNH_2_ and TMSOTf as a key transformation.

The preparation of solasodine is outlined in Scheme 2. The key transformation in our method was a simultaneous opening of F-ring of diosgenin with a Lewis acid and introduction of a nitrogen atom as a carbamate group in position 26. We commenced our studies with an investigation of the reaction of diosgenin pivalate with benzyl carbamate in the presence of TMSOTf. In the initial attempt diosgenin pivalate (**1**) was treated with TMSOTf and CbzNH_2_ at room temperature to provide the desired 26-benzyloxycarbonylaminopseudodiosgenin derivative **2** in 52% yield and the corresponding 26-benzyloxycarbonylaminolactol **3** in 6% yield. The latter product was easily converted into **2** by heating in dry toluene in presence of 4 Å molecular sieves in 82% yield.

In order to find the best reaction conditions for preparation of steroidal 26-carbamate, diosgenin reactions with various carbamates (benzyl, methyl, *t*-butyl) in presence of different Lewis acids (TMSOTf, BF_3_xOEt_2_, Tf_2_NH, TiCl_4_) were studied (Table 1).

**Scheme 3:**
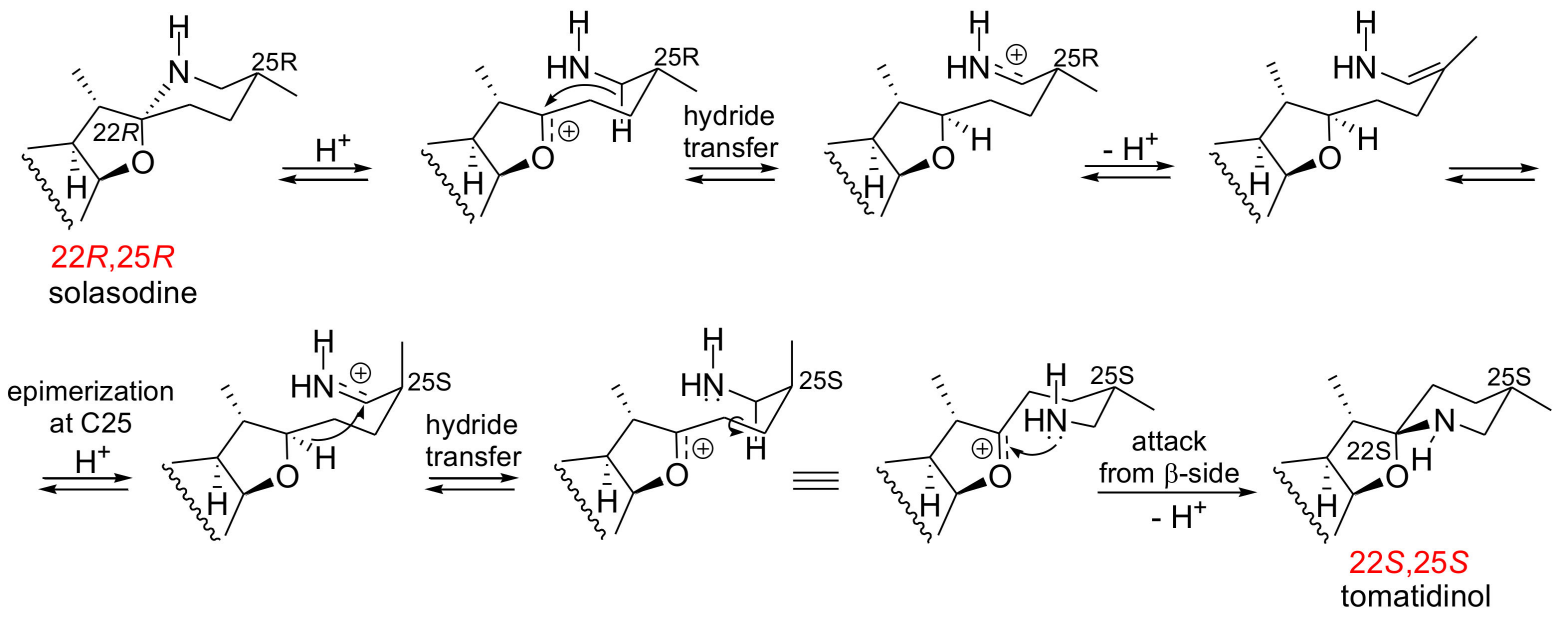
Mechanism of solasodine-tomatidinol equilibration in acid medium.

The use of other Lewis acids, e.g., BF_3_xOEt_2_, Tf_2_NH, or TiCl_4_, as reaction promotors led to decreased product yields due to formation of a more complex mixture of products than in reactions with TMSOTf or a marginal conversion, as in case of TiCl_4_ (Table 1, entries 3–5). Also replacing DCM by benzene did not improve the reaction efficiency (Table 1, entry 2). The reaction of diosgenin pivalate with methyl carbamate promoted by TMSOTf yielded 26-methoxycarbonylamino- pseudodiosgenin **2a** and 26-methoxycarbonylaminolactol **3a** in 36% and 20% yield, respectively (Table 1, entry 6). In the case of diosgenin pivalate reactions with *t*-butyl carbamate we observed, probably due to steric hindrance by the bulky *t*-butyl group, only a very low substrate conversion (Table 1, entries 7 and 8). The optimization experiments proved that the initially applied reaction conditions (TMSOTf, CbzNH_2_, DCM, r.t.) were the most effective (Table 1, entry 1). The key intermediate **2** was obtained in 57% overall yield for two steps including the F-ring opening and dehydration of byproduct **3** (Scheme 2). Having elaborated the direct route to steroidal 26-carbamate **2** from diosgenin pivalate, we established its use for the solasodine synthesis. Having in mind a facile conversion of **2** to solasodine, we looked for acidic conditions which could allow for deprotection of the 26-amino group and simultaneous rebuilding of the F-ring. Among all reagents (TMSI [25,26,27], TBAF [28], chloroborocatechol [29], TfOH [30], BF_3_xOEt_2_/Me_2_S [31,32], AcBr/BuOH [33]) attempted for this transformation only the latter one, dry HBr formed *in situ* from AcBr and butanol, gave the desired solasodine derivative **4** in good yield (53%). Analogously to Tian’s method [22], we also isolated 5,6-dehydrotomatidine **5** (tomatidinol) as a byproduct in 10% yield. The formation of this solasodine 22S,25S-isomer **5** was caused by a reversible hydride transfer process from C26 to C22 in the oxocarbenium ion formed by protonation of 26-aminopseudodiosgenin and a subsequent imine-enamine tautomerization (Scheme 3) resulting in a change of the stereogenic center configurations at C22 and C25 [22]. It should be noted that in both diastereomers, solasodine and tomatidinol, the methyl group at C25 occupies the equatorial position. During the entire process the (*S*) configuration at C20 remains intact.

To simplify the solasodine preparation, after completion of diosgenin pivalate reaction with TMSOTf and CbzNH_2_, the solvent (DCM) was evaporated and the crude reaction product, after standard work-up, without purification or separation, was dissolved in butanol and treated with AcBr. Using this protocol, solasodine and tomatidinol pivalates (**4** and **5**) were isolated in significantly improved overall yields, 45% and 8%, respectively. A comparison of this synthesis of solasodine with other methods recently described in literature [21,22,23] is provided in Table S1 (see Supplementary Materials).

## 3. Materials and Methods 

### 3.1. General 

NMR spectra were recorded with an Avance II 400 spectrometer (Bruker, Fällanden, Switzerland) operating at 400 MHz (^1^H) or 100 MHz (^13^C) using CDCl_3_ solutions with TMS as the internal standard (only selected signals from the ^1^H-NMR spectra are reported). Coupling constants (*J*) are given in Hz. Infrared spectra were recorded using Attenuated Total Reflectance (ATR) (Madison, Wi, USA) as solid samples with a Series II Magna-IR 550 FT-IR spectrometer (Nicolet, Madison, Wi, USA). Mass spectra were obtained at 70 eV with an AMD-604 spectrometer (Agilent, Santa Clara, NJ, USA). The reaction products were isolated by column chromatography, performed using 70-230 mesh silica gel (J.T. Baker, Phillipsburg, NJ, USA). 

### 3.2. Chemical Synthesis

#### 3.2.1. Typical Procedure for the Reaction between Diosgenin Pivalate and Carbamate in the Presence of Lewis Acid

To a solution of diosgenin pivalate (**1**, 0.2 g, 0.4 mmol, 1 equiv.) and CbzNH_2_ (0.127 g, 0.84 mmol, 2.2 equiv.) in DCM TMSOTf (0.14 mL, 0.8 mmol, 2 equiv.) was added. The reaction mixture was stirred at r.t. for 16 h. After this time, the mixture was poured into aqueous NaHCO_3_ and the product was extracted with DCM. The extract was washed with water, dried over anhydrous sodium sulfate, and the solvent was evaporated. The crude products **2** and **3** were separated by column chromatography using silica gel as adsorbent and hexane/ethyl acetate as eluent. Products **2a** and **3a** were obtained according to a similar procedure using methyl carbamate instead of benzyl carbamate.

*26-Benzyloxycarbonylaminopseudodiosgenin pivalate* (**2**) eluted with hexane/EtOAc (94:6): amorphous solid, R_f_ = 0.25 (hexane/EtOAc 7:3). ^1^H-NMR δ 7.36 (m, 5H), 5.39 (m, 1H), 5.10 (s, 2H), 4.84 (m, 1H), 4.74 (m, 1H), 4.58 (m, 1H), 3.10 (m, 2H), 2.47 (dd, *J_1_* = 10.2, *J_2_* = 0.0, 1H), 2.31 (m, 2H), 1.59 (s, 3H), 1.19 (s, 9H), 1.05 (s, 3H), 0.92 (d, *J* = 6.6, 3H), 0.68 (s, 3H). ^13^C-NMR δ 177.8 (C), 156.6 (C), 151.9 (C), 151.8 (C), 140.2 (C), 137.1 (C), 128.5 (2CH), 128.02 (CH), 127.99 (2CH), 122.2 (CH), 103.7 (C), 84.5 (CH), 73.6 (CH), 66.7 (CH_2_), 64.6 (CH), 55.3 (CH), 50.4 (CH), 39.8 (CH_2_), 38.7 (C), 38.2 (CH_2_), 37.2 (CH_2_), 36.9 (C), 34.2 (CH_2_), 33.4 (CH), 32.3 (CH_2_), 31.7 (CH_2_), 31.6 (CH_2_), 31.5 (CH), 27.8 (CH_2_), 27.2 (3CH_3_), 23.3 (CH_2_), 21.2 (CH_2_), 19.4 (CH_3_), 17.5 (CH_3_), 14.0 (CH_3_), 11.5 (CH_3_). IR (ATR): ν_max_ (cm^−1^): 3359, 1725, 1532, 1456, 1286, 1163. ESI-MS 632 [M + H]^+^. HRMS calcd for C_40_H_58_NO_5_ 632.4310 (M + H)^+^, found 632.4309.

*(25R)-26-Benzyloxycarbonylamino-22ξ-hydroxyfurost-5-en-3β-yl pivalate* (**3**) eluted with hexane/EtOAc (75:25): amorphous solid, R_f_ = 0.11 (hexane/AcOEt 7:3). ^1^H-NMR δ 7.36 (m, 5H), 5.38 (m, 1H), 5.10 (s, 2H), 4.91 (m, 1H), 4.60 (m, 2H), 3.12 (m, 2H), 2.60 (m, 1H), 1.20 (s, 9H), 1.06 (s, 3H), 0.92 (d, *J* = 7.2, 3H), 0.90 (d, *J* = 6.8, 3H), 0.81 (s, 3H). ^13^C-NMR δ 178.0 (C), 156.7 (C), 139.8 (C), 136.6 (C), 128.5 (3CH), 128.1 (2CH), 122.2 (CH), 110.3 (C), 81.4 (CH), 73.4 (CH), 66.7 (CH_2_), 62.7 (CH), 56.4 (CH), 50.0 (CH), 40.6 (C), 40.14 (CH), 40.08 (CH), 39.6 (CH_2_), 38.6 (C), 38.0 (CH_2_), 37.0 (CH_2_), 36.7 (C), 33.7 (CH), 31.9 (CH_2_), 31.8 (CH_2_), 29.7 (CH_2_), 29.3 (CH_2_), 27.6 (CH_2_), 27.1 (3CH_3_), 22.7 (CH_2_), 20.8 (CH_2_), 19.4 (CH_3_), 17.5 (CH_3_), 15.5 (CH_3_), 14.1 (CH_3_). IR (ATR): ν_max_ (cm^−1^): 3372, 1712, 1526, 1447, 1280, 1237, 1153, 1021. ESI-MS 1321 (2M + Na)^+^, 632 (M + H − H_2_O)^+^. HRMS calcd for C_80_H_118_N_2_O_12_Na 1321.8577 (2M + Na)^+^, found 1321.8518.

*26-Methoxycarbonylaminopseudodiosgenin pivalate* (**2a**) eluted with hexane/EtOAc (9:1): amorphous solid, R_f_ = 0.4 (hexane/AcOEt 7:3). ^1^H-NMR δ 5.38 (m, 1H), 4.73 (m, 1H, H-16), 4.58 (m, 1H), 3.66 (s, 3H), 3.07 (m, 2H), 2.48 (dd, *J_1_* = 10.2, *J_2_* = 0.0, 1H), 1.59 (s, 3H), 1.19 (s, 9H), 1.05 (s, 3H), 0.91 (d, *J* = 6.7, 3H), 0.69 (s, 3H). ^13^C-NMR δ 178.0 (C), 157.2 (C), 151.4 (C), 139.8 (C), 122.1 (CH), 103.9 (C), 84.3 (CH), 73.4 (CH), 64.2 (CH), 55.0 (CH), 52.0 (CH_3_), 50.0 (CH), 43.3 (C), 39.5 (CH_2_), 38.6 (C), 38.0 (CH_2_), 37.0 (CH_2_), 36.7 (C), 34.1 (CH_2_), 32.2 (CH_2_), 31.4 (CH_2_), 31.3 (CH_2_, CH), 31.2 (CH), 27.6 (CH_2_), 27.1 (3CH_3_), 23.2 (CH_2_), 21.0 (CH_2_), 19.4 (CH_3_), 13.94 (CH_3_), 13.90 (CH_3_), 11.6 (CH_3_). IR (ATR): ν_max_ (cm^−1^): 3373, 1711, 1532, 1450, 1360, 1159, 1022. ESI-MS 1133 (2M + Na)^+^, 556 (M + H)^+^. HRMS calcd for C_34_H_54_NO_5_ 556.43997 (M + H)^+^, found 556.4004.

*(25R)-26-Methoxycarbonylamino-22ξ-hydroxyfurost-5-en-3β-yl pivalate* (**3a**) eluted with hexane/EtOAc (60:40): amorphous solid, R_f_ = 0.11 (hexane/EtOAc 7:3). ^1^H-NMR δ 5.38 (m, 1H), 4.79 (m, 1H), 4.59 (m, 2H), 3.67 (s, 3H), 3.09 (m, 2H), 2.31 (m, 2H), 1.19 (s, 9H), 1.06 (s, 3H), 1.03 (d, *J* = 8.5, 3H), 0.92 (d, *J* = 6.6, 3H), 0.81 (s, 3H). ^13^C-NMR δ 178.0 (C), 157.3 (C), 139.8 (C), 122.2 (CH), 110.3 (C), 81.4 (CH), 73.4 (CH), 62.6 (CH), 56.4 (CH), 52.1 (CH_3_), 49.9 (CH), 40.6 (C), 40.2 (CH) 39.6 (CH_2_), 38.6 (C), 38.0 (CH_2_), 37.0 (CH_2_), 36.7 (C), 36.6 (CH_2_), 33.8 (CH), 32.0 (CH_2_), 31.8 (CH_2_), 31.4 (CH), 29.7 (CH_2_), 27.6 (2CH_2_), 27.1 (3CH_3_), 20.8 (CH_2_), 19.4 (CH_3_), 16.2 (CH_3_), 15.6 (CH_3_), 15.5 (CH_3_). IR (ATR): ν_max_ (cm^−1^): 3360, 1710, 1523, 1444, 1381, 1286, 1158, 1033. ESI-MS 1369 (2M + Na)^+^, 574 (M + H)^+^. HRMS calcd for C_34_H_56_NO_6_ 574.4102 (M + H)^+^, found 574.4112.

#### 3.2.2. Deprotection of the 26-amino Group with AcBr/BuOH

Acetyl bromide (0.012 mL, 0.16 mmol, 4 equiv.) was added to the stirred, ice-cold-solution of 26-benzyloxycarbonylaminopseudodiosgenin pivalate (**2**, 25 mg, 0.04 mmol, 1 equiv.) in dry BuOH (10 mL). The reaction mixture was heated under reflux overnight. The solvent was evaporated in vacuo. The residue was dissolved in CHCl_3_ and treated with 5 M NaOH aq (2 mL). The two-phase mixture was intensively stirred for 30 min. Then the organic layer was separated, washed with brine and water, dried over Na_2_SO_4_ and concentrated in vacuo. The crude products **4** and **5** were separated by silica gel column chromatography. 

*Solasodine pivalate* (**4**) eluted with hexane/EtOAc (65:35–50:50): colorless crystals, m.p.: 211–213 °C (MeOH/CHCl_3_), R_f_ = 0.21 (hexane/EtOAc 4:6). ^1^H-NMR δ 5.38 (m, 1H), 4.58 (m, 1H), 4.28 (m, 1H), 2.64 (m, 1H), 2.31 (m, 2H), 2.02 (m, 2H), 1.19 (s, 9H), 1.06 (s, 3H), 0.96 (d, *J* = 7.0, 3H), 0.86 (d, *J* = 6.2, 3H), 0.82 (s, 3H). ^13^C-NMR δ 178.0 (C), 139.9 (C), 122.2 (CH), 98.3 (C), 78.6 (CH), 73.4 (CH), 62.8 (CH), 56.4 (CH), 50.0 (CH), 47.6 (CH_2_), 41.2 (CH), 40.5 (C), 39.9 (CH_2_), 38.6 (C), 38.0 (CH_2_), 37.0 (CH_2_), 36.8 (C), 34.1 (CH_2_), 32.2 (CH_2_), 32.1 (CH_2_), 31.4 (2CH), 30.3 (CH_2_), 27.6 (CH_2_), 27.1 (3CH_3_), 20.9 (CH_2_), 19.4 (CH_3_), 19.3 (CH_3_), 16.4 (CH_3_), 15.3 (CH_3_). IR (ATR): ν_max_ (cm^–1^): 3341, 1713, 1470, 1372, 1289, 1174. ESI-MS 498 [M + H]^+^. HRMS calcd for C_32_H_52_NO_3_ 498.3942 (M + H)^+^, found 498.3954.

*5,6-Dehydrotomatidine pivalate* (**5**) eluted with hexane/EtOAc (88:12–84:16): colorless crystals, m.p.: 202–203 °C (MeOH/CHCl_3_), R_f_ = 0.41 (hexane/EtOAc 4:6). ^1^H-NMR δ 5.38 (m, 1H), 4.58 (m, 1H), 4.15 (m, 1H), 2.74 (m, 1H), 2.31 (m, 2H), 2.01 (m, 2H), 1.19 (s, 9H), 1.06 (s, 3H), 0.99 (d, *J* = 6.6, 3H), 0.87 (d, *J* = 6.6, 3H), 0.86 (s, 3H). ^13^C-NMR δ 178.0 (C), 139.9 (C), 122.2 (CH), 99.1 (C), 78.5 (CH), 73.4 (CH), 61.9 (CH), 55.9 (CH), 50.2 (CH_2_), 50.0 (CH), 43.0 (CH), 40.6 (C), 39.9 (CH_2_), 38.6 (C), 38.0 (CH_2_), 37.0 (CH_2_), 36.8 (C), 32.8 (CH_2_), 32.1 (CH_2_), 31.4 (CH), 31.0 (CH), 28.5 (CH_2_), 27.6 (CH_2_), 27.1 (3CH_3_), 26.6 (CH_2_), 20.8 (CH_2_), 19.4 (CH_3_), 19.3 (CH_3_), 16.7 (CH_3_), 15.9 (CH_3_). IR (ATR): ν_max_ (cm^−1^): 3413, 1713, 1457, 1378, 1279, 1151. ESI-MS 498 [M + H]^+^. HRMS calcd for C_32_H_52_NO_3_ 498.3942 (M + H)^+^, found 498.3945.

#### 3.2.3. Synthesis of Solasodine Pivalate from Diosgenin Pivalate

To a solution of diosgenin pivalate (**1**, 0.09 g, 0.18 mmol, 1 equiv.) and CbzNH_2_ (0.054 g, 0.36 mmol, 2 equiv.) in DCM TMSOTf (0.063 mL, 0.35 mmol, 1.9 equiv.) was added. Reaction mixture was stirred at r.t. for 16 h. After this time, the reaction mixture was poured into aqueous NaHCO_3_ and the product was extracted with DCM. The extract was washed with water, dried over anhydrous sodium sulfate, and the solvent was evaporated. The residue was dissolved in dry butanol (10 mL), cooled to 0 °C and treated with acetyl bromide (0.3 mL). The reaction mixture was heated under reflux overnight. The solvent was evaporated in vacuo. The residue was dissolved in CHCl_3_ and treated with 5 M NaOH aq (3 mL). The two-phase mixture was intensively stirred for 30 min. Then the organic layer was separated, washed with water and dried. The solvent was evaporated in vacuo and reaction products were separated by silica-gel column chromatography. Tomatidinol pivalate (**5**, 0.007 g, 8%) was eluted with hexane/EtOAc (88:12–84:16) followed by solasodine pivalate (**4**, 0.04 g, 45%) eluted with hexane/EtOAc (65:35–50:50). 

## 4. Conclusions

To summarize, the results of our preliminary studies towards the development of an alternative procedure for the synthesis of solasodine are presented herein. A two-step protocol for solasodine pivalate synthesis from diosgenin pivalate is proposed. The new approach involves a sequence of two simultaneous transformations: firstly, the F-ring opening and 26-benzyloxycarbonyloamination proceeding during the reaction of diosgenin pivalate with TMSOTf and CbzNH_2_ and secondly, deprotection of the 26-amino group and *N*-cyclization by treatment the intermediate carbamate with HBr generated *in situ* from the AcBr reaction with butanol. The desired solasodine derivative was obtained in 45% yield, in addition to its 22*S*,25*S*-isomer, tomatidenol, which was isolated in 8% yield. Further optimization in terms of yield, conditions, scalability, as well as further study of the last deprotection step (pivalate removal) are still needed.

## Figures and Tables

**Table 1:** Study of diosgenin pivalate reactions with carbamates in presence of Lewis acids.

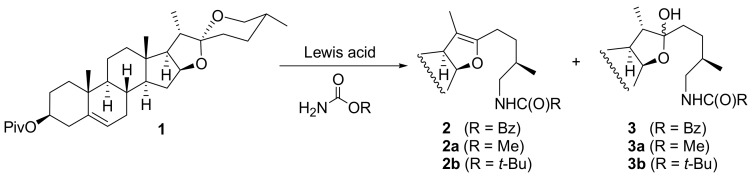					
Entry	Lewis Acid (Equiv.)	Carbamate (Equiv.)	Solvent	Temp., Time	Product (Yield)
1	TMSOTf (2)	R = Bn (2.2)	DCM	r.t., 16h	**2** (52%), **3** (6%)
2	TMSOTf (2)	R = Bn (2.2)	benzene	40 °C, 16h	**2** (20%), **3** (10%)
3	BF3xOEt2 (4)	R = Bn (4)	DCM	r.t., 16h	**2** (35%), **3** (15%)
4	Tf2NH (0.2)	R = Bn (1.5)	DCM	r.t., 16h	**2** (20%), **3** (20%)
5	TiCl4 (2)	R = Bn (2.2)	DCM	r.t., 16h	**2** (0%), **3** (0%)
6	TMSOTf (2)	R = Me (2.2)	DCM	r.t., 16h	**2a** (36%), **3a** (20%)
7	TMSOTf (2)	R = t-Bu (2.2)	DCM	r.t.-reflux, 48 h	conversion < 10%1
8	BF3xOEt2 (4)	R = t-Bu (4)	DCM	r.t., 16h	conversion < 10%1

^1^ The reaction products were not isolated from the reaction mixture because of low substrate conversion.

## Supplementary Materials

The following are available online at https://www.mdpi.com/1420-3049/24/6/1132/s1, Table S1: A comparison of this synthesis of solasodine with other methods recently described in literature [21,22,23] and Figures S2–S18: ^1^H-NMR, ^13^C-NMR, and DEPT spectra of compounds **2**–**5**.
