# Supplementary material for: Two-step Synthesis of Solasodine Pivalate from Diosgenin Pivalate
Source: Molecules. 2019 Mar 21;24(6):1132. doi: 10.3390/molecules24061132 (PMC6471385; doi:10.3390/molecules24061132)
Supplement: Supplementary file 1 [file molecules-24-01132-s001.pdf]

Supplementary Materials for

Two-step synthesis of solasodine pivalate from diosgenin pivalate

Agnieszka Wojtkielewicz *, Urszula Kiełczewska and Jacek W. Morzycki

Institute of Chemistry, University of Białystok, K. Ciołkowskiego 1K, 15-245 Białystok, Poland;
a.wojtkielewicz@uwb.edu.pl; ulakielczewska@interia.eu; morzycki@uwb.edu.pl
*Correspondence: a.wojtkielewicz@uwb.edu.pl; Tel.: +48-85-738-8043

Table of contents

Table S1. Comparison of prices and hazards of reagents used in three recent syntheses of solasodine………………..2

Spectra of compound **2** (^{1}H NMR, ^{13}C NMR)……………………………………………………………………….3

Spectra of compound **3** (^{1}H NMR, ^{13}C NMR) ………………………………………………………………………6

Spectra of compound **2a** (^{1}H NMR, ^{13}C NMR) ……………………………………………………………………..9

Spectra of compound **3a** (^{1}H NMR, ^{13}C NMR) ……………………………………………………………………12

Spectra of compound **4** (^{1}H NMR, ^{13}C NMR) ……………………………………………………………………...14

Spectra of compound **5** (^{1}H NMR, ^{13}C NMR) ……………………………………………………………………...17

Table S1. Comparison of prices and hazards of reagents used in three recent syntheses of solasodine.

Reference no. in manuscript	Number of steps	Total yield of product	Reagents (equiv, price*, hazards#)
This paper	2	45% (solasodine pivalate)	**TMSOTf** (2 equiv. 1.6 €/g, H226, H314)<br>**$CbzNH_2$** (2.2. equiv. 0.9 €/g, According to SDS#: Not a hazardous substance or mixture according to Regulation (EC) No. 1272/2008. Not a hazardous substance or mixture according to EC-directives 67/548/EEC or 1999/45/EC.)<br>**AcBr** (4 – 22 equiv. 0.3 €/g, H314)<br>**BuOH** (58.60 €/l, H226, H315, H318, H335, H336)
**21**	7	57% (solasodine)	**TFAT** (1.5 equiv. 42.6 €/g, H301, H314)<br>**Ac_2O** (5 equiv. 0.016 €/g, H226, H302, H314, H330)<br>**Pyridine** (10 equiv. 0.17 €/g, H225, H302+H312+H332, H315, H319))<br>**TsCl** (2 equiv. 0.068 €/g, H290, H315, H317, H318)<br>**NaN_3** (3 equiv. 0.195 €/g, H300+H310, H373, H410),<br>**TMSCl** (2 equiv. 1.06 €/g, H225, H301+H331, H312, H315, H318)<br>**NaI** (2 equiv. 2.36 €/g, H315, H319, H400)
**22**	3	50% (solasodine)	**BF_3xOEt_2** (5 equiv. 0.22 €/g, H226, H302, H314, H330, H372)<br>**LiBr** (8 equiv. 0.4 €/g, H302, H315, H317, H319),<br>**NaN_3** (2 equiv. 0.195 €/g, H300+H310, H373, H410),<br>**DMF** (146 €/l, H226, H312+H332, H319, H360D),<br>**TMSCl** (2.2 equiv. 1.06 €/g, H225, H301+H331, H312, H315, H318)<br>**NaI** (2 equiv. 2.36 €/g, H315, H319, H400)
**23**	3	43% (solasodine)	**BF_3xOEt_2** (4 equiv. 0.22 €/g, H226, H302, H314, H330, H372)<br>***p*-$TsNH_2$** (4 equiv. 0.065 €/g, Not a hazardous substance or mixture according to Regulation (EC) No. 1272/2008.),<br>**Na** (10 equiv. 1 €/g, H260, H314),<br>**naphtalene** (100 equiv., 0.05 €/g, H228, H302, H351, H410),<br>**TMSCl** (6.3 equiv. 1.06 €/g, H225, H301+H331, H312, H315, H318)

*Price from Sigma Aldrich catalogue (https://www.sigmaaldrich.com/catalog/search).
#SDS found on website: https://www.sigmaaldrich.com/catalog/product/aldrich/b18200?lang=en&region=GB

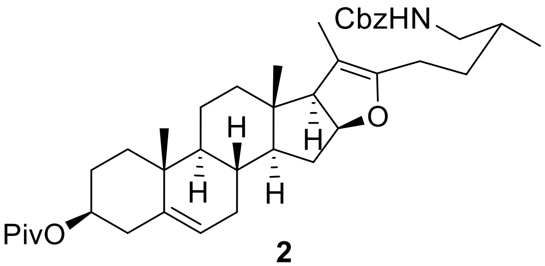

CbzHN
PivO
H
H
H
H
O

2

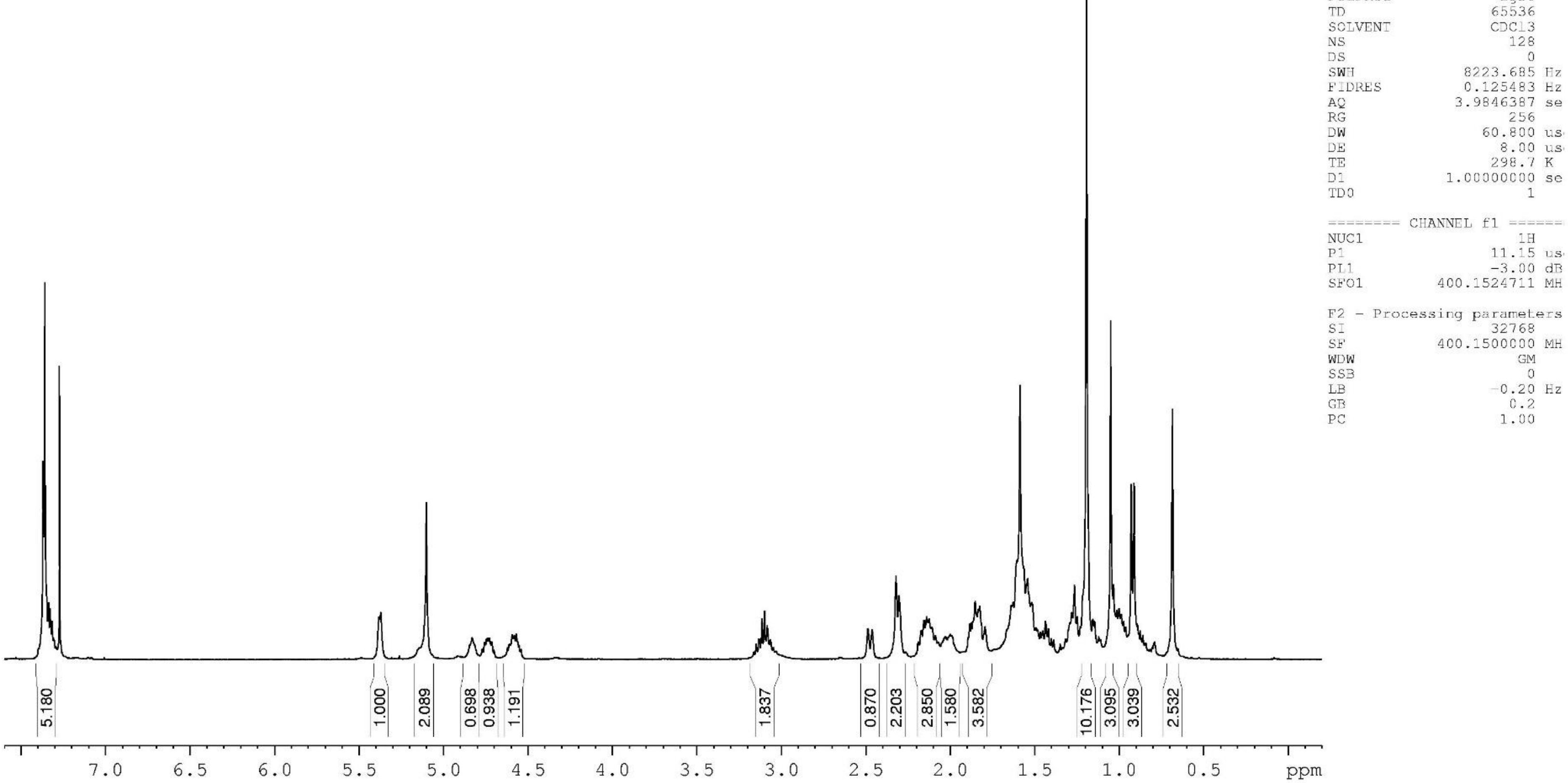

Current Data Parameters
NAME AW G277rj(3)
EXPNO 1
PROCNO 1
F2 - Acquisition Parameter
Date_ 20170731
Time 16.20
INSTRUM spect
PROBHD 5 mm PABBO BB-
PULPROG zg30
TD 65536
SOLVENT CDCl3
NS 128
DS 0
SWH 8223.685 Hz
FIDRES 0.125483 Hz
AQ 3.9846387 se
RG 256
DW 60.800 us
DE 8.00 us
TE 298.7 K
D1 1.00000000 se
TD0 1
======== CHANNEL f1 ======
NUC1 1H
P1 11.15 us
PL1 -3.00 dB
SFO1 400.1524711 MH
F2 - Processing parameters
SI 32768
SF 400.1500000 MH
WDW GM
SSB 0
LB -0.20 Hz
GB 0.2
PC 1.00
5.180
1.000
2.089
0.698
0.938
1.191
1.837
0.870
2.203
2.850
1.580
3.582
10.176
3.095
3.039
2.532
7.0
6.5
6.0
5.5
5.0
4.5
4.0
3.5
3.0
2.5
2.0
1.5
1.0
0.5
ppm

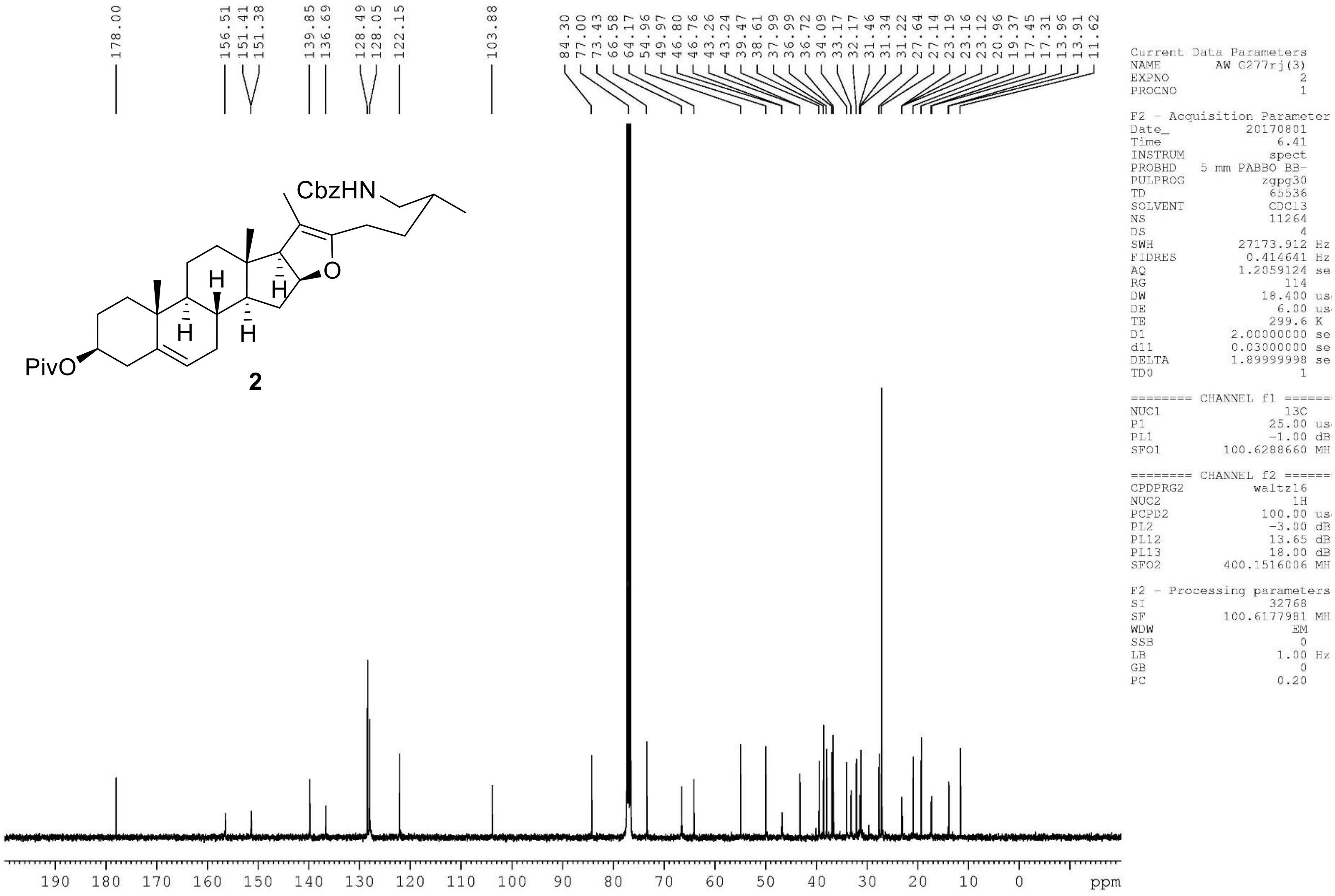
178.00
156.51
151.41
151.38
139.85
136.69
128.49
128.05
122.15
103.88
84.30
77.00
73.43
66.58
64.17
54.96
49.97
46.80
46.76
43.26
43.24
39.47
38.61
37.99
36.99
36.72
34.09
33.17
32.17
31.46
31.34
31.22
27.64
27.14
23.19
23.16
23.12
20.96
19.37
17.45
17.31
13.96
13.91
11.62
CbzHN
H
H
O
H
H
PivO
2
Current Data Parameters
NAME AW G277rj(3)
EXPNO 2
PROCNO 1
F2 - Acquisition Parameter
Date_ 20170801
Time 6.41
INSTRUM spect
PROBHD 5 mm PABBO BB-
PULPROG zgpg30
TD 65536
SOLVENT CDCl3
NS 11264
DS 4
SWH 27173.912 Hz
FIDRES 0.414641 Hz
AQ 1.2059124 se
RG 114
DW 18.400 us
DE 6.00 us
TE 299.6 K
D1 2.00000000 se
d11 0.03000000 se
DELTA 1.89999998 se
TD0 1
======== CHANNEL f1 ======
NUC1 13C
P1 25.00 us
PL1 -1.00 dB
SFO1 100.6288660 MH
======== CHANNEL f2 ======
CPDPRG2 waltz16
NUC2 1H
PCPD2 100.00 us
PL2 -3.00 dB
PL12 13.65 dB
PL13 18.00 dB
SFO2 400.1516006 MH
F2 - Processing parameters
SI 32768
SF 100.6177981 MH
WDW EM
SSB 0
LB 1.00 Hz
GB 0
PC 0.20
190 180 170 160 150 140 130 120 110 100 90 80 70 60 50 40 30 20 10 0 ppm

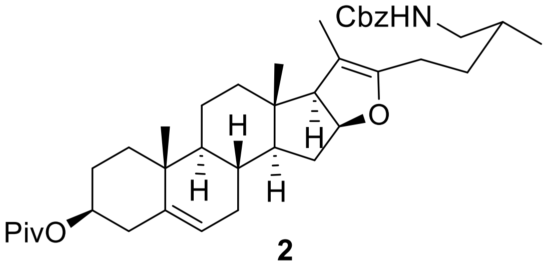

CbzHN
O
H
H
H
H
PivO

2

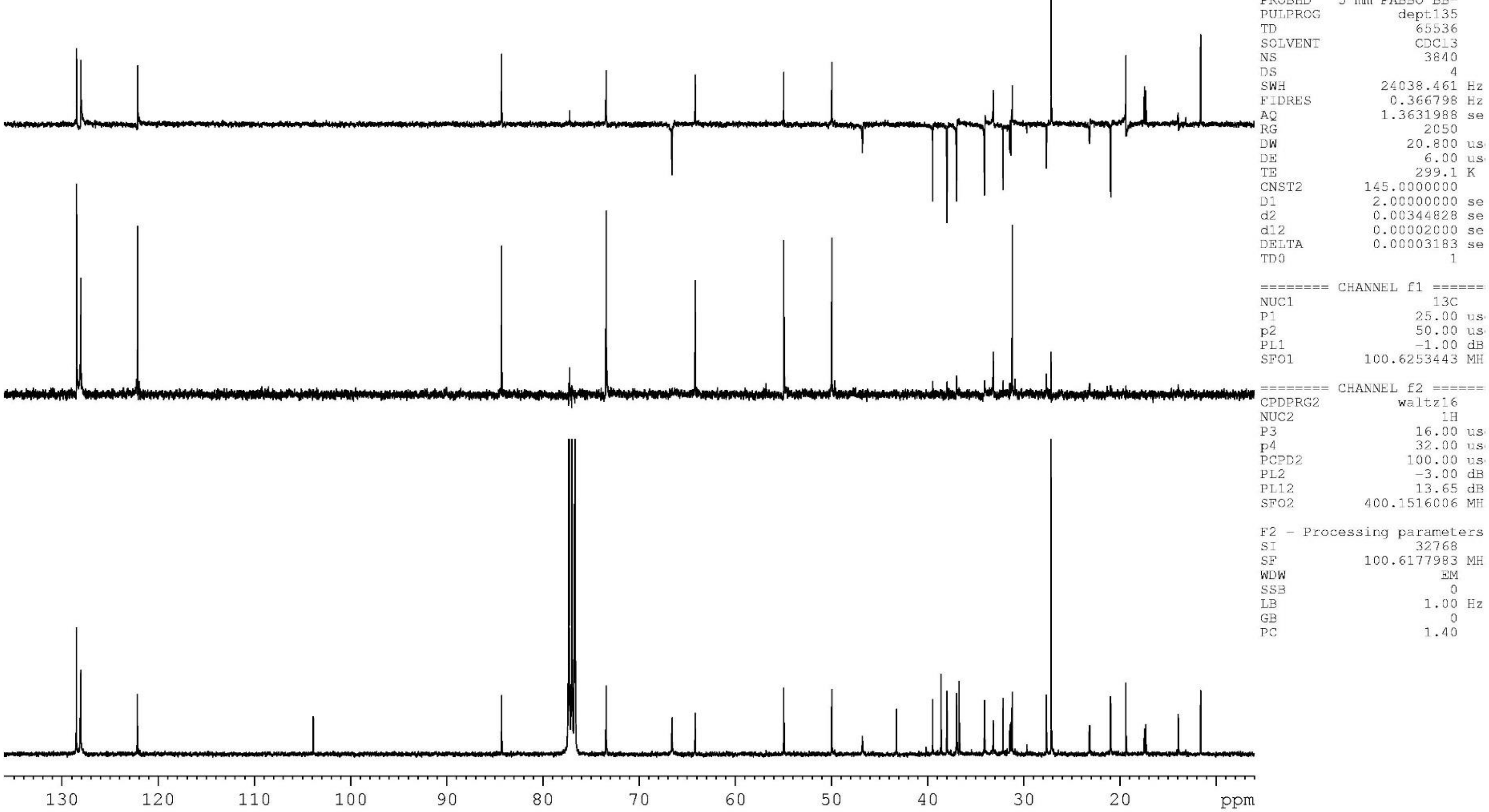

Current Data Parameters
NAME AW G277rj(3)
EXPNO 4
PROCNO 1
F2 - Acquisition Parameter
Date_ 20170801
Time 10.21
INSTRUM spect
PROBHD 5 mm PABBO BB-
PULPROG dept135
TD 65536
SOLVENT CDCl3
NS 3840
DS 4
SWH 24038.461 Hz
FIDRES 0.366798 Hz
AQ 1.3631988 se
RG 2050
DW 20.800 us
DE 6.00 us
TE 299.1 K
CNST2 145.0000000
D1 2.00000000 se
d2 0.00344828 se
d12 0.00002000 se
DELTA 0.00003183 se
TD0 1
======== CHANNEL f1 ======
NUC1 13C
P1 25.00 us
p2 50.00 us
PL1 -1.00 dB
SFO1 100.6253443 MH
======== CHANNEL f2 ======
CPDPRG2 waltz16
NUC2 1H
P3 16.00 us
p4 32.00 us
PCPD2 100.00 us
PL2 -3.00 dB
PL12 13.65 dB
SFO2 400.1516006 MH
F2 - Processing parameters
SI 32768
SF 100.6177983 MH
WDW EM
SSB 0
LB 1.00 Hz
GB 0
PC 1.40
130
120
110
100
90
80
70
60
50
40
30
20
ppm

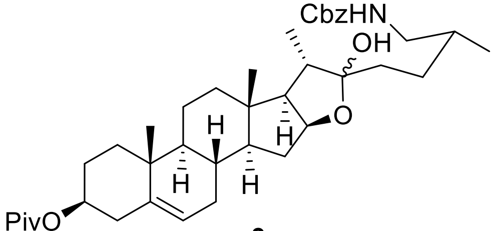
CbzHN
OH
H
H
H
H
O
PivO

3

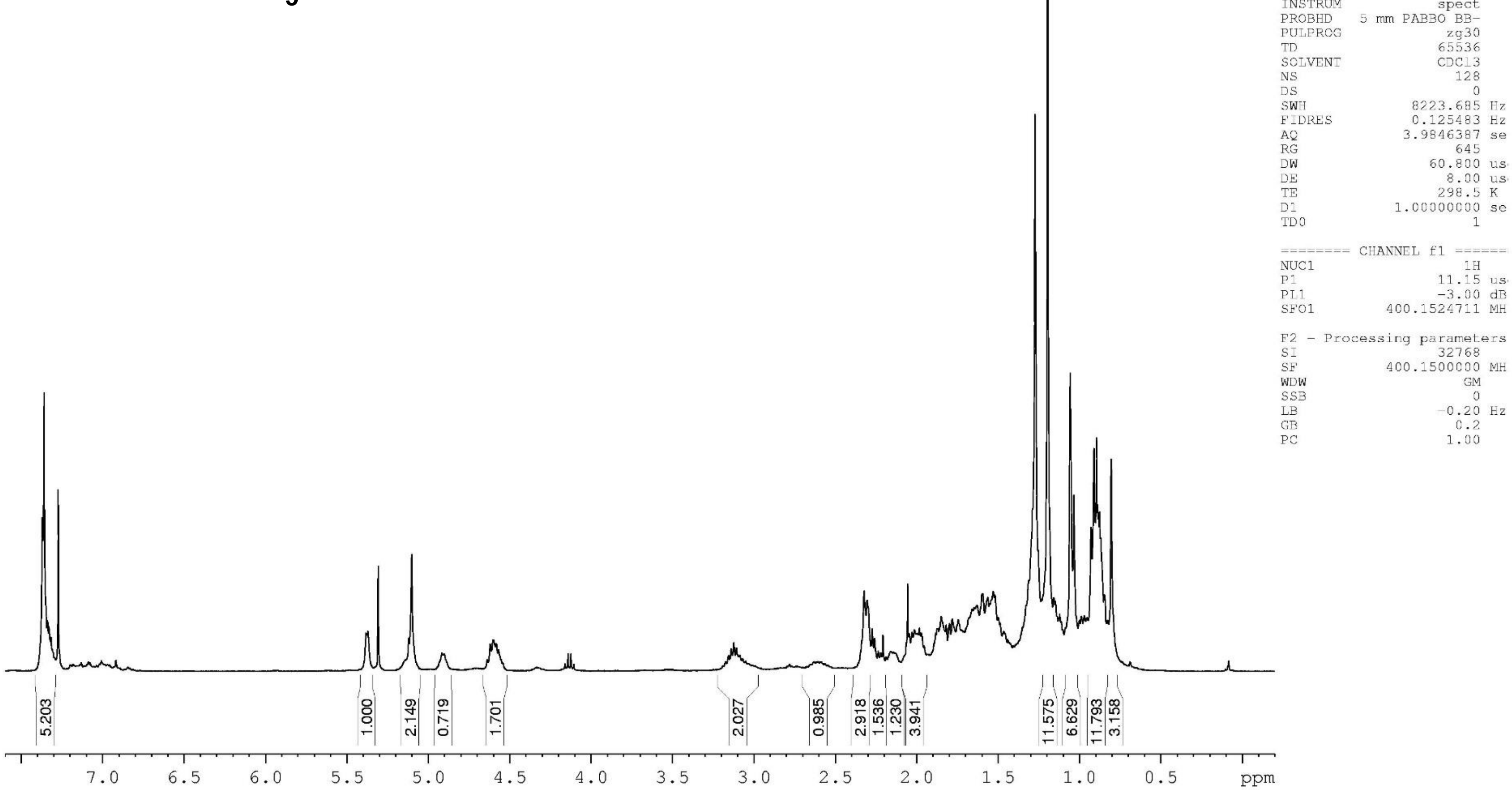
Current Data Parameters
NAME AW G277'(8)
EXPNO 1
PROCNO 1
F2 - Acquisition Parameter
Date_ 20170425
Time 15.26
INSTRUM spect
PROBHD 5 mm PABBO BB-
PULPROG zg30
TD 65536
SOLVENT CDCl3
NS 128
DS 0
SWH 8223.685 Hz
FIDRES 0.125483 Hz
AQ 3.9846387 se
RG 645
DW 60.800 us
DE 8.00 us
TE 298.5 K
D1 1.00000000 se
TD0 1
======== CHANNEL f1 ======
NUC1 1H
P1 11.15 us
PL1 -3.00 dB
SFO1 400.1524711 MH
F2 - Processing parameters
SI 32768
SF 400.1500000 MH
WDW GM
SSB 0
LB -0.20 Hz
GB 0.2
PC 1.00
5.203
1.000
2.149
0.719
1.701
2.027
0.985
2.918
1.536
1.230
3.941
11.575
6.629
11.793
3.158
7.0
6.5
6.0
5.5
5.0
4.5
4.0
3.5
3.0
2.5
2.0
1.5
1.0
0.5
ppm

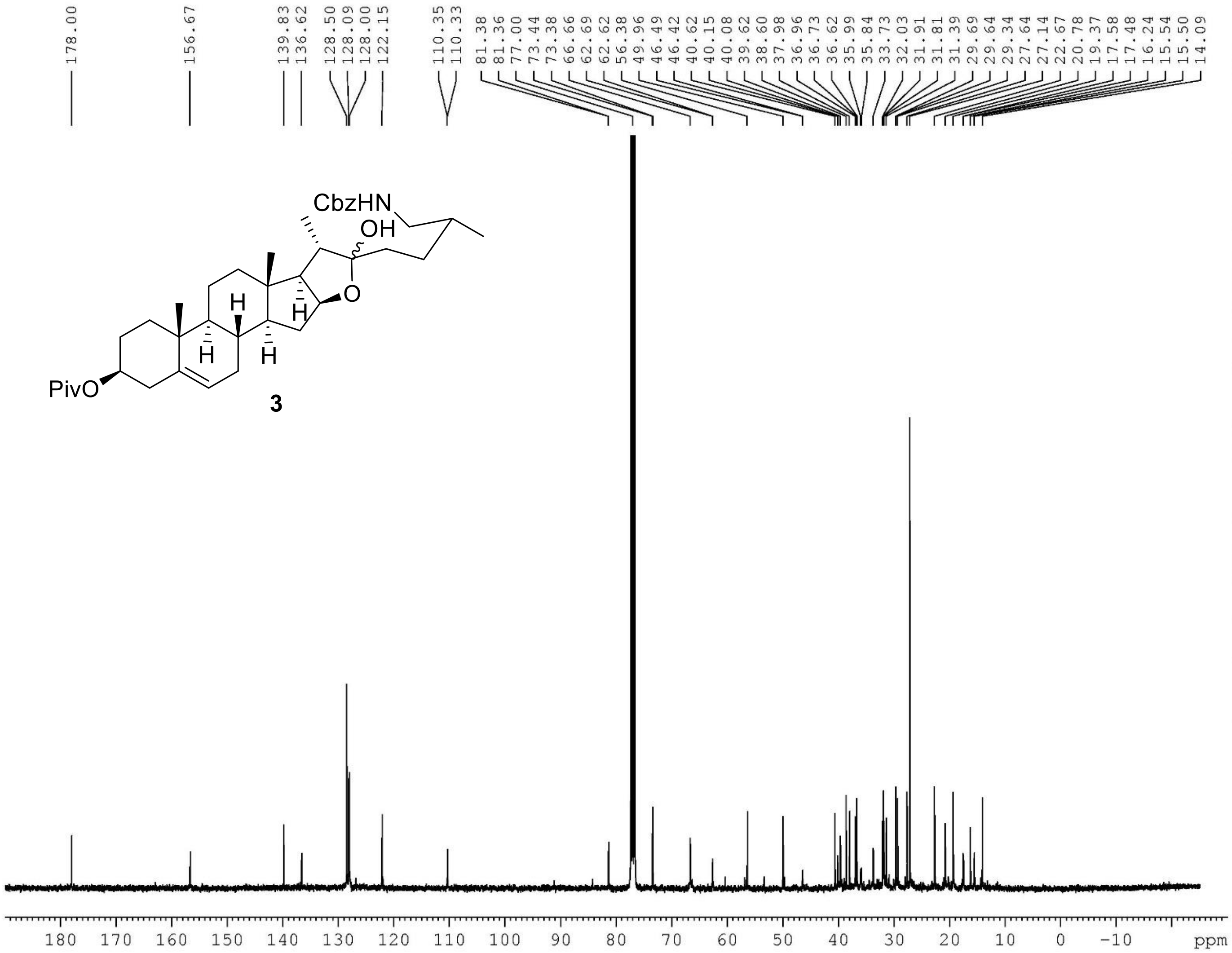


```
Current Data Parameters
NAME          AW G277'(B)
EXPNO                   2
PROCNO                  1

F2 - Acquisition Parameter
Date_            20170427
Time                20.09
INSTRUM             spect
PROBHD   5 mm PABBO BB-
PULPROG           zgpg30
TD                 65536
SOLVENT            CDCl3
NS                 12800
DS                     4
SWH            27173.912 Hz
FIDRES          0.414641 Hz
AQ             1.2059124 se
RG                   161
DW                18.400 us
DE                  6.00 us
TE                 299.1 K
D1            2.00000000 se
d11           0.03000000 se
DELTA         1.89999998 se
TD0                    1

======== CHANNEL f1 ======
NUC1                 13C
P1                 25.00 us
PL1                -1.00 dB
SFO1         100.6288660 MH

======== CHANNEL f2 ======
CPDPRG2           waltz16
NUC2                   1H
PCPD2             100.00 us
PL2                -3.00 dB
PL12               13.65 dB
PL13               18.00 dB
SFO2         400.1516006 MH

F2 - Processing parameters
SI                 32768
SF           100.6177984 MH
WDW                   EM
SSB                    0
LB                  1.00 Hz
GB                     0
PC                  0.20
```

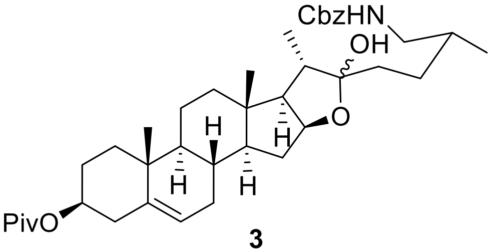
CbzHN
OH
O
H
H
H
H
PivO

3

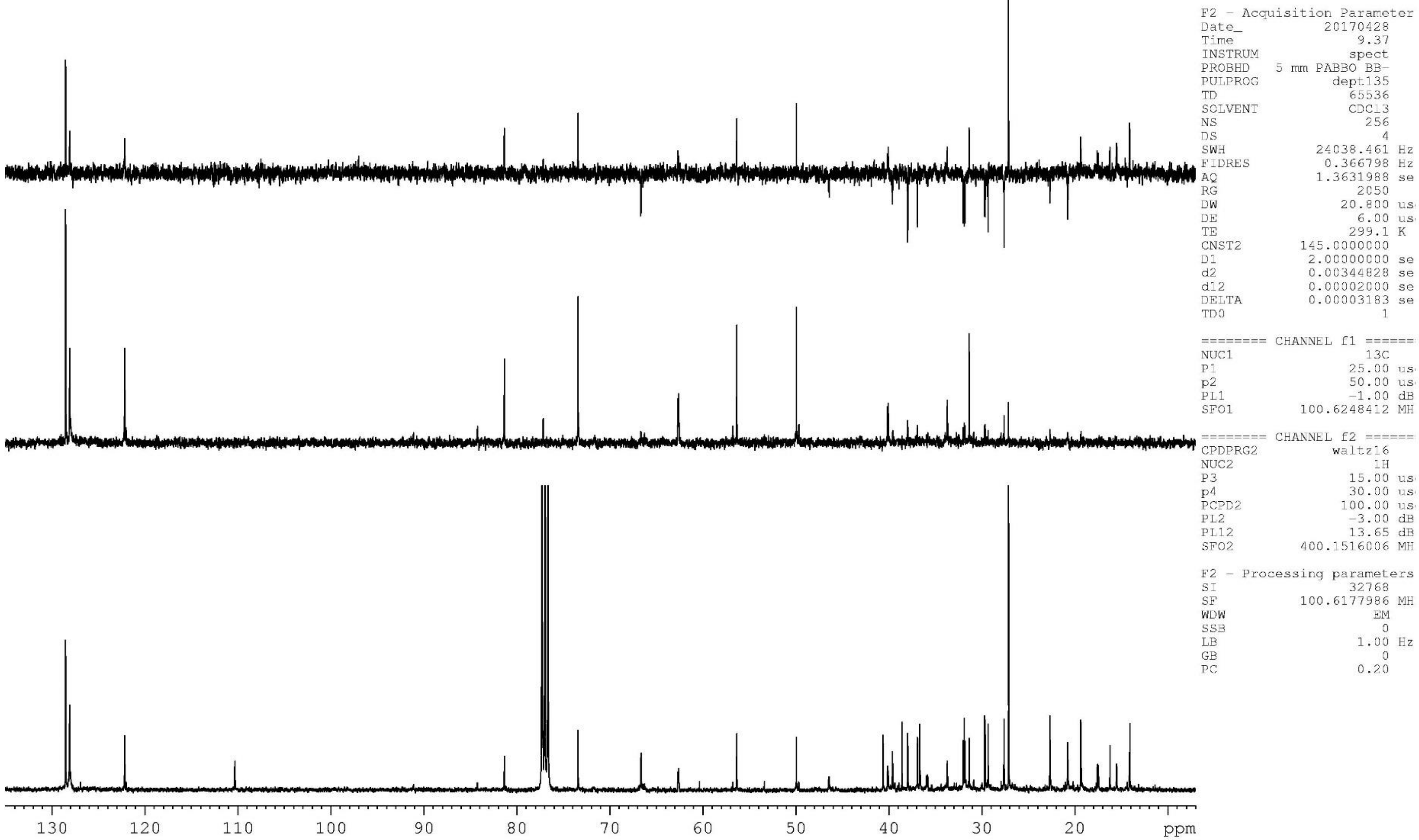
Current Data Parameters
NAME AW G277'(B)
EXPNO 4
PROCNO 1
F2 - Acquisition Parameter
Date_ 20170428
Time 9.37
INSTRUM spect
PROBHD 5 mm PABBO BB-
PULPROG dept135
TD 65536
SOLVENT CDCl3
NS 256
DS 4
SWH 24038.461 Hz
FIDRES 0.366798 Hz
AQ 1.3631988 se
RG 2050
DW 20.800 us
DE 6.00 us
TE 299.1 K
CNST2 145.0000000
D1 2.00000000 se
d2 0.00344828 se
d12 0.00002000 se
DELTA 0.00003183 se
TD0 1
======== CHANNEL f1 ======
NUC1 13C
P1 25.00 us
p2 50.00 us
PL1 -1.00 dB
SFO1 100.6248412 MH
======== CHANNEL f2 ======
CPDPRG2 waltz16
NUC2 1H
P3 15.00 us
p4 30.00 us
PCPD2 100.00 us
PL2 -3.00 dB
PL12 13.65 dB
SFO2 400.1516006 MH
F2 - Processing parameters
SI 32768
SF 100.6177986 MH
WDW EM
SSB 0
LB 1.00 Hz
GB 0
PC 0.20
130 120 110 100 90 80 70 60 50 40 30 20 ppm

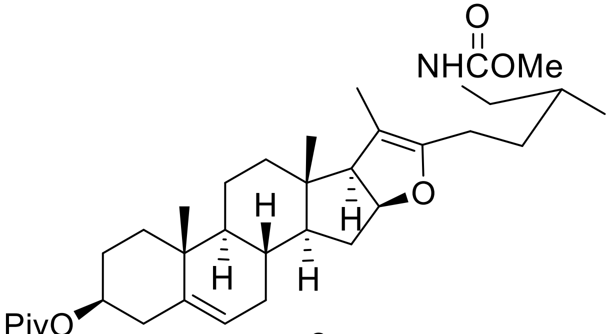
NHCOMe
PivO

2a

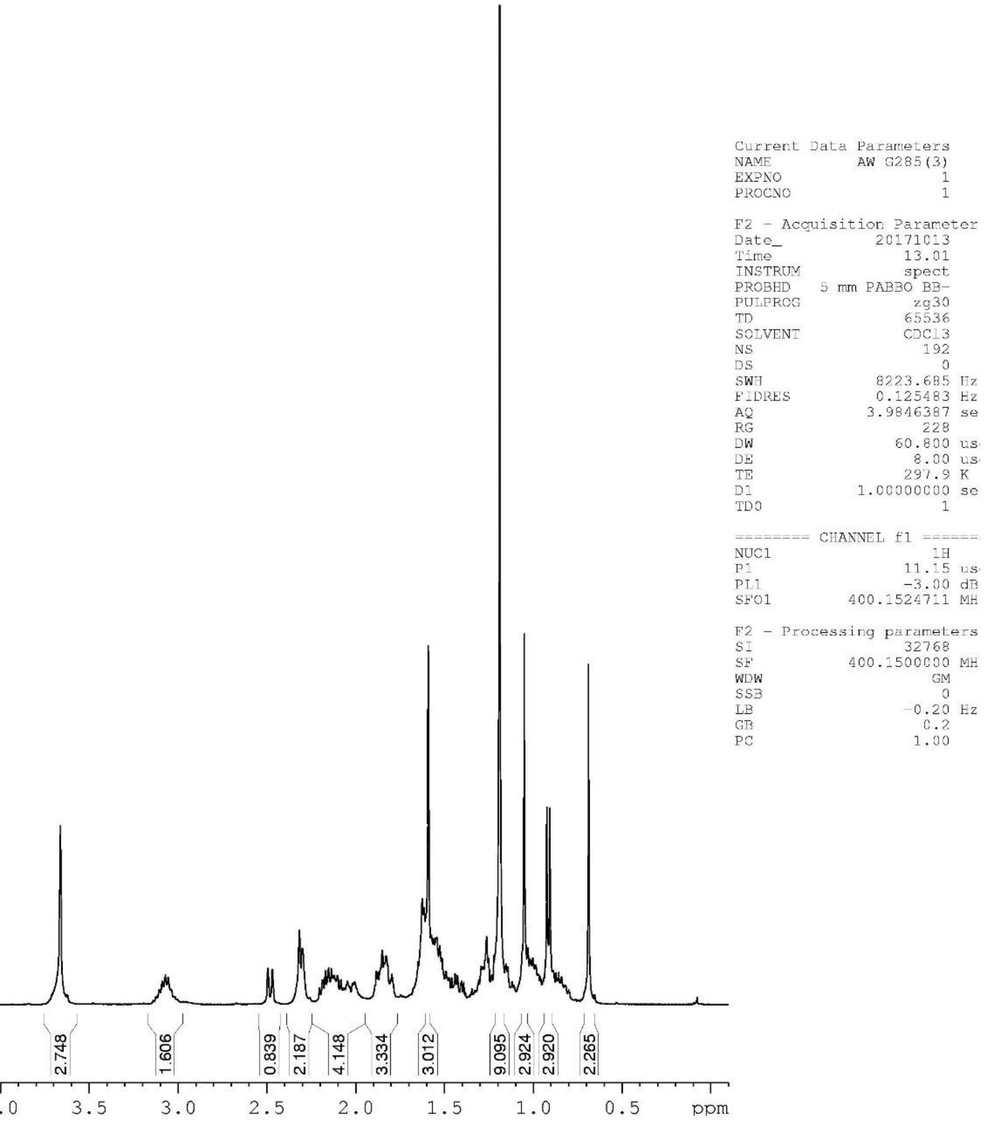
Current Data Parameters
NAME AW G285(3)
EXPNO 1
PROCNO 1
F2 - Acquisition Parameter
Date_ 20171013
Time 13.01
INSTRUM spect
PROBHD 5 mm PABBO BB-
PULPROG zg30
TD 65536
SOLVENT CDCl3
NS 192
DS 0
SWH 8223.685 Hz
FIDRES 0.125483 Hz
AQ 3.9846387 se
RG 228
DW 60.800 us
DE 8.00 us
TE 297.9 K
D1 1.00000000 se
TD0 1
======== CHANNEL f1 ======
NUC1 1H
P1 11.15 us
PL1 -3.00 dB
SFO1 400.1524711 MH
F2 - Processing parameters
SI 32768
SF 400.1500000 MH
WDW GM
SSB 0
LB -0.20 Hz
GB 0.2
PC 1.00
1.000
1.412
1.114
2.748
1.606
0.839
2.187
4.148
3.334
3.012
9.095
2.924
2.920
2.265
7.0
6.5
6.0
5.5
5.0
4.5
4.0
3.5
3.0
2.5
2.0
1.5
1.0
0.5
ppm

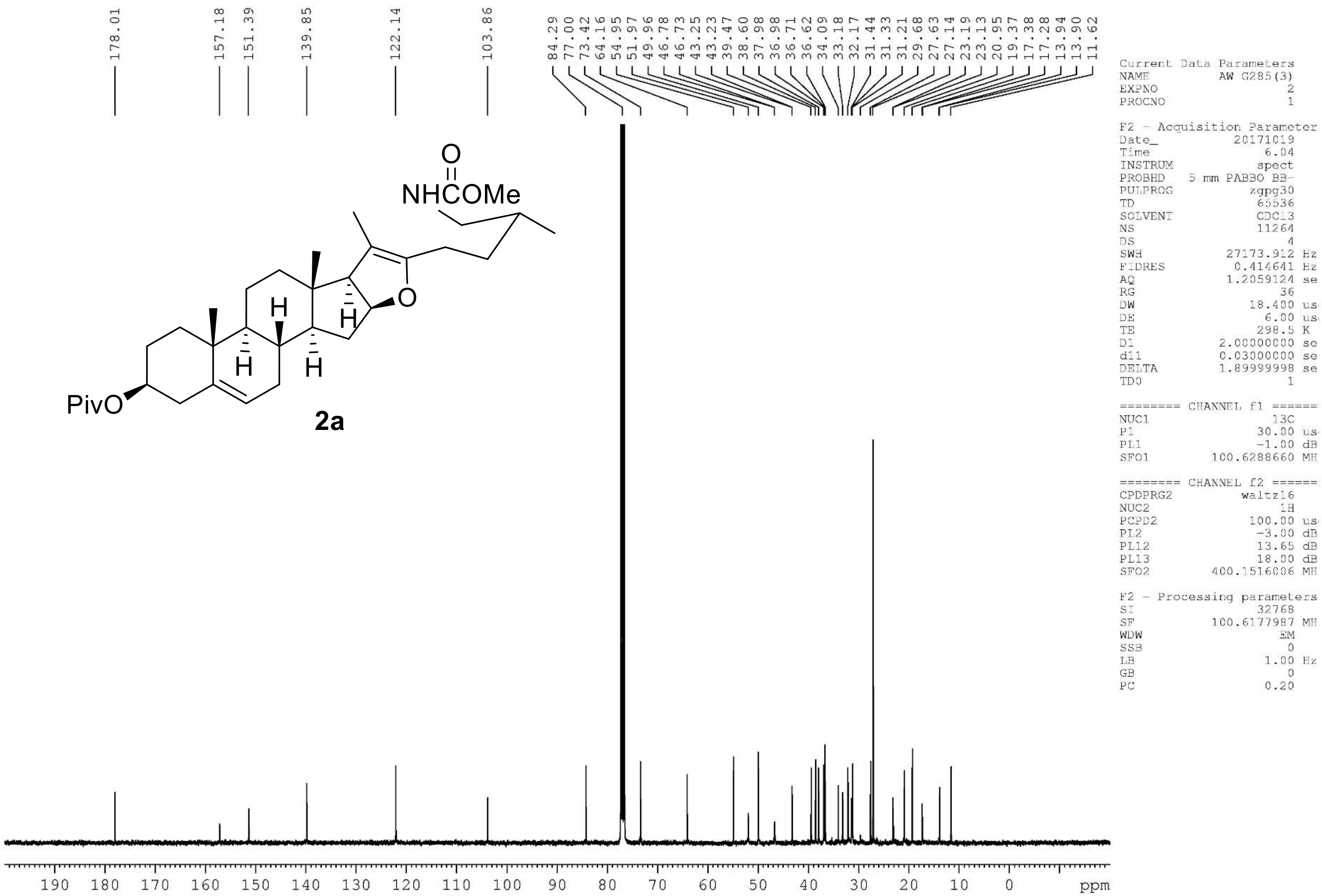
178.01
157.18
151.39
139.85
122.14
103.86
84.29
77.00
73.42
64.16
54.95
51.97
49.96
46.78
46.73
43.25
43.23
39.47
38.60
37.98
36.98
36.71
36.62
34.09
33.18
32.17
31.44
31.33
31.21
29.68
27.63
27.14
23.19
23.13
20.95
19.37
17.38
17.28
13.94
13.90
11.62
O
NHCOMe
H
H
H
H
PivO
2a
190 180 170 160 150 140 130 120 110 100 90 80 70 60 50 40 30 20 10 0 ppm
Current Data Parameters
NAME AW G285(3)
EXPNO 2
PROCNO 1
F2 - Acquisition Parameter
Date_ 20171019
Time 6.04
INSTRUM spect
PROBHD 5 mm PABBO BB-
PULPROG zgpg30
TD 65536
SOLVENT CDCl3
NS 11264
DS 4
SWH 27173.912 Hz
FIDRES 0.414641 Hz
AQ 1.2059124 se
RG 36
DW 18.400 us
DE 6.00 us
TE 298.5 K
D1 2.00000000 se
d11 0.03000000 se
DELTA 1.89999998 se
TD0 1
======== CHANNEL f1 ======
NUC1 13C
P1 30.00 us
PL1 -1.00 dB
SFO1 100.6288660 MH
======== CHANNEL f2 ======
CPDPRG2 waltz16
NUC2 1H
PCPD2 100.00 us
PL2 -3.00 dB
PL12 13.65 dB
PL13 18.00 dB
SFO2 400.1516006 MH
F2 - Processing parameters
SI 32768
SF 100.6177987 MH
WDW EM
SSB 0
LB 1.00 Hz
GB 0
PC 0.20

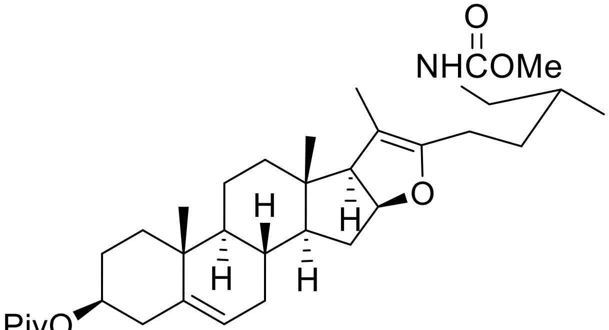

NHCOMe
O
H
H
H
H
PivO

2a

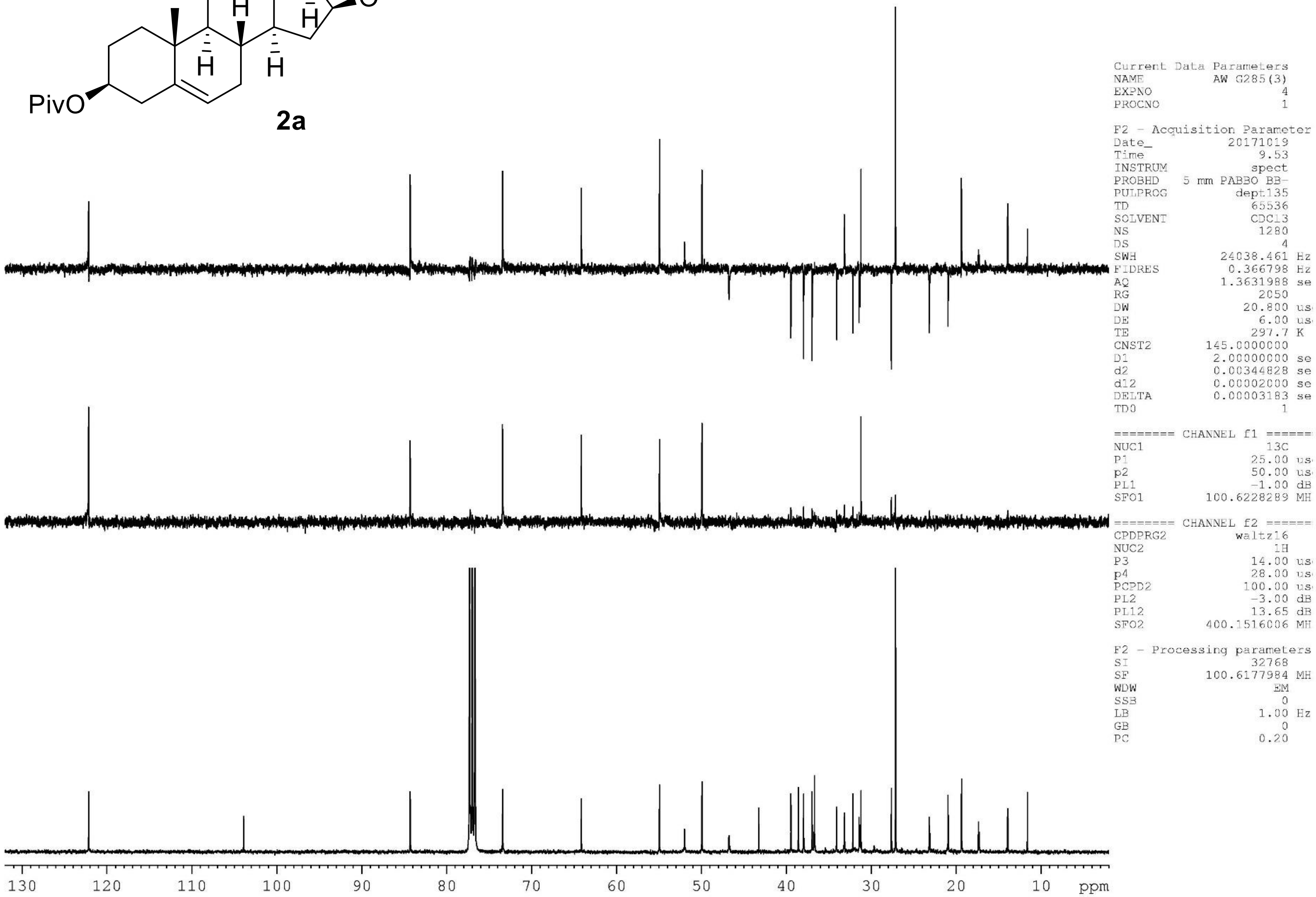

Current Data Parameters
NAME AW G285(3)
EXPNO 4
PROCNO 1
F2 - Acquisition Parameter
Date_ 20171019
Time 9.53
INSTRUM spect
PROBHD 5 mm PABBO BB-
PULPROG dept135
TD 65536
SOLVENT CDCl3
NS 1280
DS 4
SWH 24038.461 Hz
FIDRES 0.366798 Hz
AQ 1.3631988 se
RG 2050
DW 20.800 us
DE 6.00 us
TE 297.7 K
CNST2 145.0000000
D1 2.00000000 se
d2 0.00344828 se
d12 0.00002000 se
DELTA 0.00003183 se
TD0 1
======== CHANNEL f1 ======
NUC1 13C
P1 25.00 us
p2 50.00 us
PL1 -1.00 dB
SFO1 100.6228289 MH
======== CHANNEL f2 ======
CPDPRG2 waltz16
NUC2 1H
P3 14.00 us
p4 28.00 us
PCPD2 100.00 us
PL2 -3.00 dB
PL12 13.65 dB
SFO2 400.1516006 MH
F2 - Processing parameters
SI 32768
SF 100.6177984 MH
WDW EM
SSB 0
LB 1.00 Hz
GB 0
PC 0.20
130 120 110 100 90 80 70 60 50 40 30 20 10 ppm

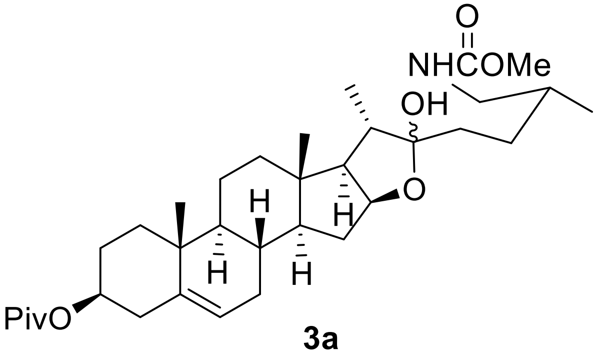

3a

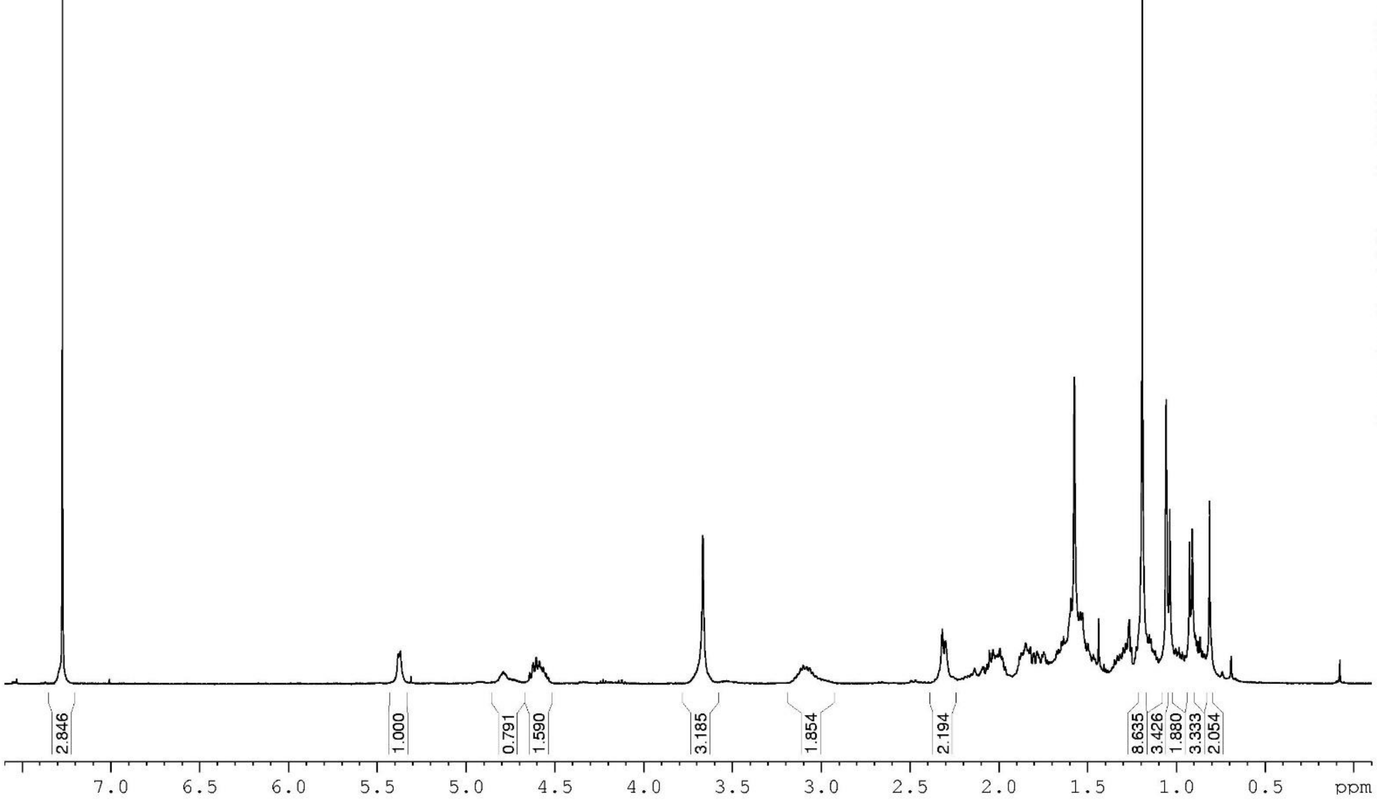

Current Data Parameters
NAME AW G285(5)
EXPNO 1
PROCNO 1

F2 - Acquisition Parameter
Date_ 20171013
Time 14.00
INSTRUM spect
PROBHD 5 mm PABBO BB-
PULPROG zg30
TD 65536
SOLVENT CDCl3
NS 192
DS 0
SWH 8223.685 Hz
FIDRES 0.125483 Hz
AQ 3.9846387 se
RG 256
DW 60.800 us
DE 8.00 us
TE 298.0 K
D1 1.00000000 se
TD0 1

======== CHANNEL f1 ======
NUC1 1H
P1 25.00 us
PL1 -3.00 dB
SFO1 400.1524711 MH

F2 - Processing parameters
SI 32768
SF 400.1500000 MH
WDW GM
SSB 0
LB -0.20 Hz
GB 0.2
PC 1.00

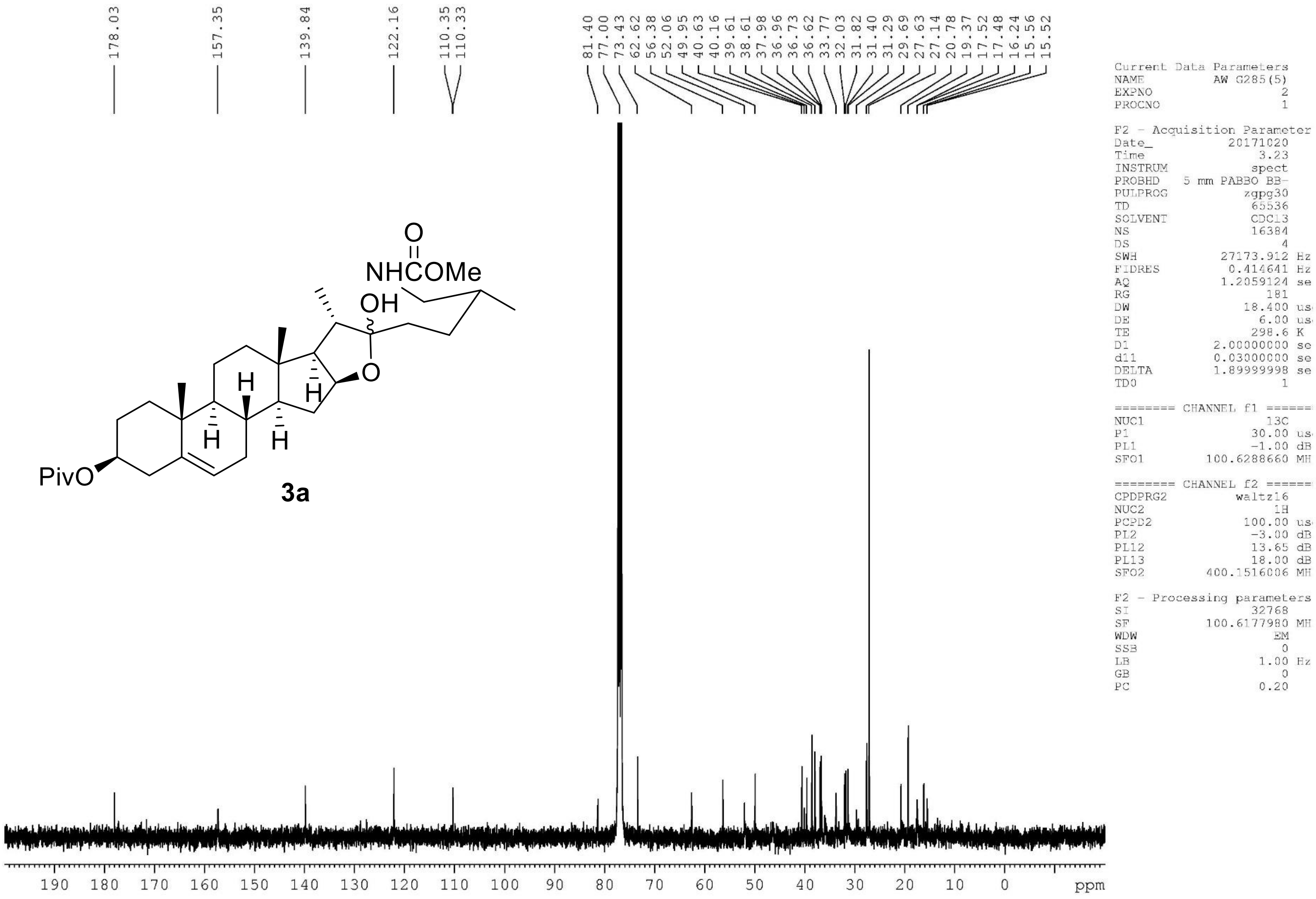
178.03
157.35
139.84
122.16
110.35
110.33
81.40
77.00
73.43
62.62
56.38
52.06
49.95
40.63
40.16
39.61
38.61
37.98
36.96
36.73
36.62
33.77
32.03
31.82
31.40
31.29
29.69
27.63
27.14
20.78
19.37
17.52
17.48
16.24
15.56
15.52
O
NHCOMe
OH
H
H
H
H
PivO
3a
Current Data Parameters
NAME AW G285(5)
EXPNO 2
PROCNO 1
F2 - Acquisition Parameter
Date_ 20171020
Time 3.23
INSTRUM spect
PROBHD 5 mm PABBO BB-
PULPROG zgpg30
TD 65536
SOLVENT CDCl3
NS 16384
DS 4
SWH 27173.912 Hz
FIDRES 0.414641 Hz
AQ 1.2059124
RG 181
DW 18.400
DE 6.00
TE 298.6 K
D1 2.00000000
d11 0.03000000
DELTA 1.89999998
TD0 1
======== CHANNEL f1 ======
NUC1 13C
P1 30.00
PL1 -1.00 dB
SFO1 100.6288660
======== CHANNEL f2 ======
CPDPRG2 waltz16
NUC2 1H
PCPD2 100.00
PL2 -3.00 dB
PL12 13.65 dB
PL13 18.00 dB
SFO2 400.1516006
F2 - Processing parameters
SI 32768
SF 100.6177980
WDW EM
SSB 0
LB 1.00 Hz
GB 0
PC 0.20
190 180 170 160 150 140 130 120 110 100 90 80 70 60 50 40 30 20 10 0 ppm

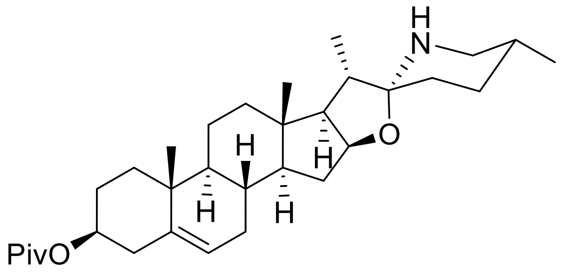

4

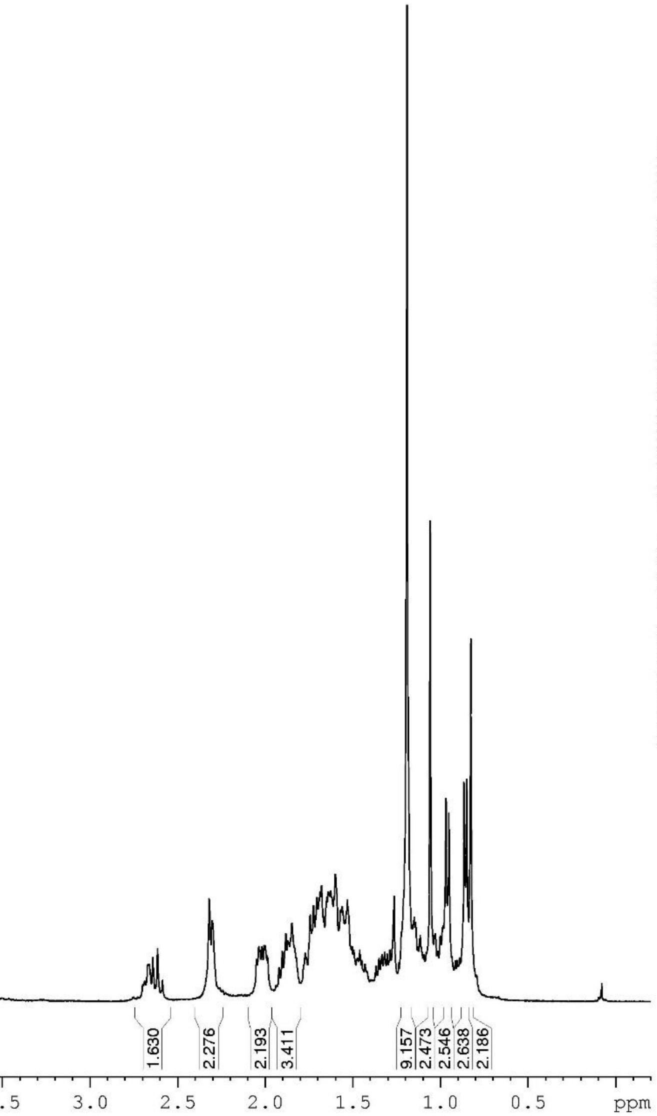


```
Current Data Parameters
NAME      AW PivSolas(3+4)
EXPNO                    1
PROCNO                   1

F2 - Acquisition Parameter
Date_             20170928
Time                 10.56
INSTRUM              spect
PROBHD   5 mm PABBO BB-
PULPROG               zg30
TD                   65536
SOLVENT              CDCl3
NS                     192
DS                       0
SWH            8223.685 Hz
FIDRES         0.125483 Hz
AQ            3.9846387 se
RG                     228
DW              60.800 us
DE                8.00 us
TE               297.5 K
D1          1.00000000 se
TD0                      1

======== CHANNEL f1 ======
NUC1                    1H
P1                25.00 us
PL1               -3.00 dB
SFO1       400.1524711 MH

F2 - Processing parameters
SI                   32768
SF         400.1500000 MH
WDW                     GM
SSB                      0
LB                -0.20 Hz
GB                     0.2
PC                    1.00
```

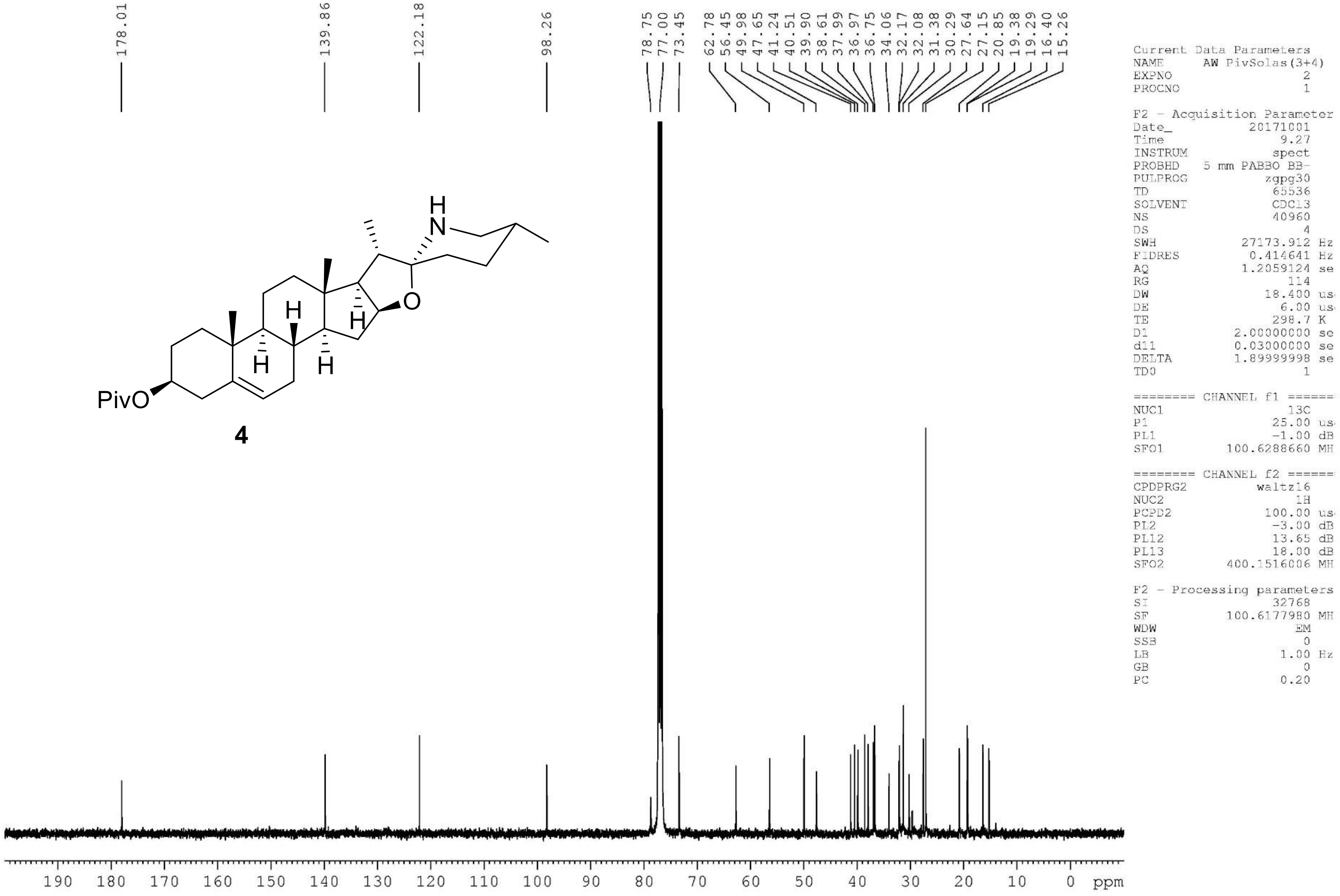

178.01
139.86
122.18
98.26
78.75
77.00
73.45
62.78
56.45
49.98
47.65
41.24
40.51
39.90
38.61
37.99
36.97
36.75
34.06
32.17
32.08
31.38
30.29
27.64
27.15
20.85
19.38
19.29
16.40
15.26
PivO
H
N
O
4
Current Data Parameters
NAME AW PivSolas(3+4)
EXPNO 2
PROCNO 1
F2 - Acquisition Parameter
Date_ 20171001
Time 9.27
INSTRUM spect
PROBHD 5 mm PABBO BB-
PULPROG zgpg30
TD 65536
SOLVENT CDCl3
NS 40960
DS 4
SWH 27173.912 Hz
FIDRES 0.414641 Hz
AQ 1.2059124 se
RG 114
DW 18.400 us
DE 6.00 us
TE 298.7 K
D1 2.00000000 se
d11 0.03000000 se
DELTA 1.89999998 se
TD0 1
======== CHANNEL f1 ======
NUC1 13C
P1 25.00 us
PL1 -1.00 dB
SFO1 100.6288660 MH
======== CHANNEL f2 ======
CPDPRG2 waltz16
NUC2 1H
PCPD2 100.00 us
PL2 -3.00 dB
PL12 13.65 dB
PL13 18.00 dB
SFO2 400.1516006 MH
F2 - Processing parameters
SI 32768
SF 100.6177980 MH
WDW EM
SSB 0
LB 1.00 Hz
GB 0
PC 0.20
190 180 170 160 150 140 130 120 110 100 90 80 70 60 50 40 30 20 10 0 ppm

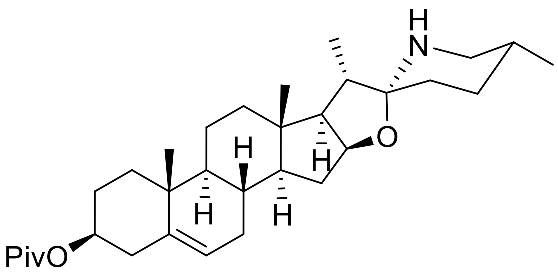
H
N
H
H
H
H
O
PivO

4

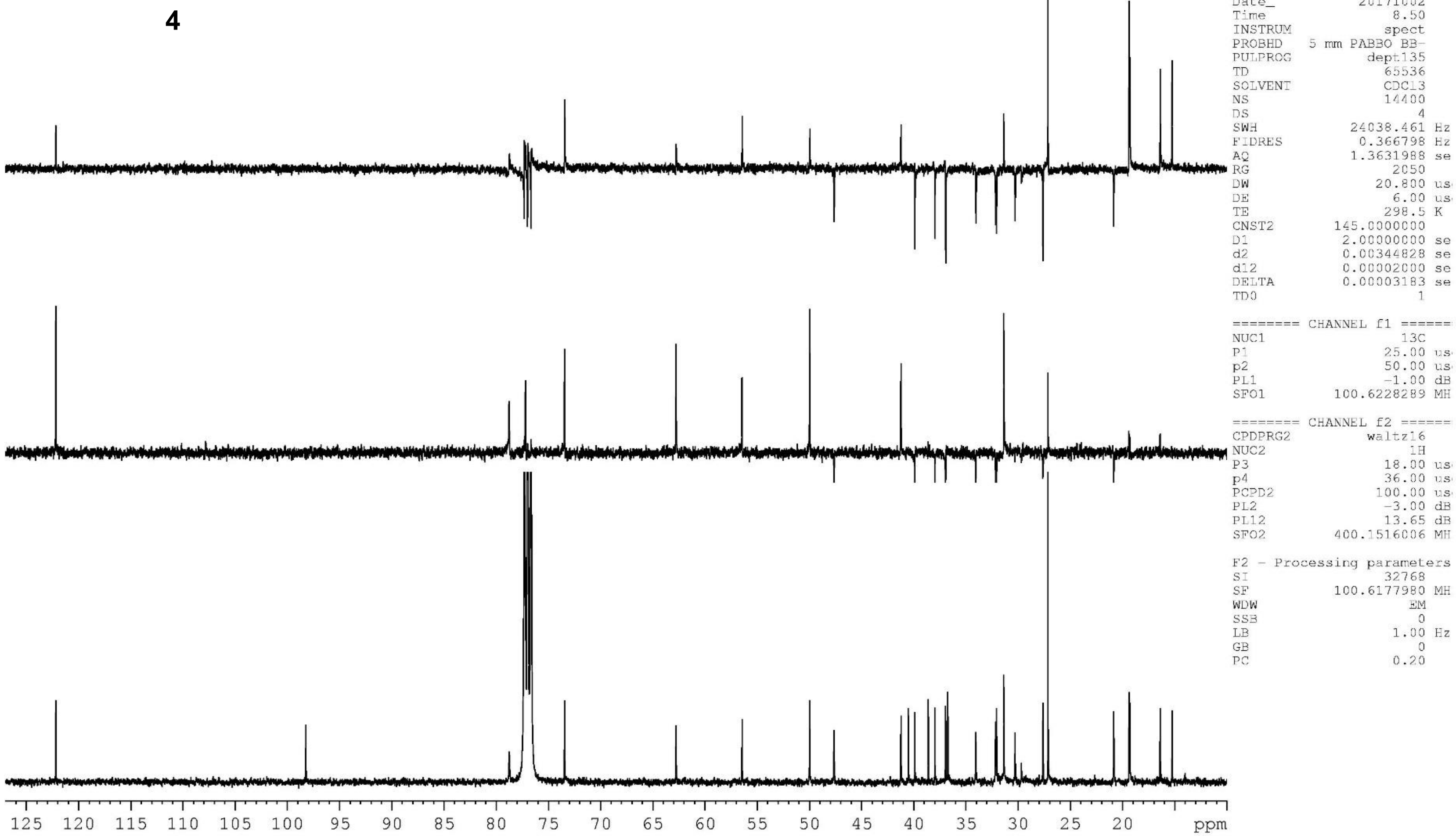
Current Data Parameters
NAME AW PivSolas(3+4)
EXPNO 4
PROCNO 1
F2 - Acquisition Parameter
Date_ 20171002
Time 8.50
INSTRUM spect
PROBHD 5 mm PABBO BB-
PULPROG dept135
TD 65536
SOLVENT CDCl3
NS 14400
DS 4
SWH 24038.461 Hz
FIDRES 0.366798 Hz
AQ 1.3631988 se
RG 2050
DW 20.800 us
DE 6.00 us
TE 298.5 K
CNST2 145.0000000
D1 2.00000000 se
d2 0.00344828 se
d12 0.00002000 se
DELTA 0.00003183 se
TD0 1
======== CHANNEL f1 ======
NUC1 13C
P1 25.00 us
p2 50.00 us
PL1 -1.00 dB
SFO1 100.6228289 MH
======== CHANNEL f2 ======
CPDPRG2 waltz16
NUC2 1H
P3 18.00 us
p4 36.00 us
PCPD2 100.00 us
PL2 -3.00 dB
PL12 13.65 dB
SFO2 400.1516006 MH
F2 - Processing parameters
SI 32768
SF 100.6177980 MH
WDW EM
SSB 0
LB 1.00 Hz
GB 0
PC 0.20
125 120 115 110 105 100 95 90 85 80 75 70 65 60 55 50 45 40 35 30 25 20 ppm

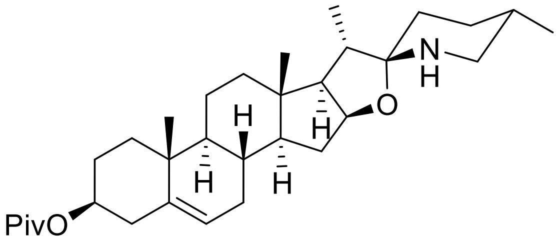
PivO
N
H
O
H
H
H
H

5

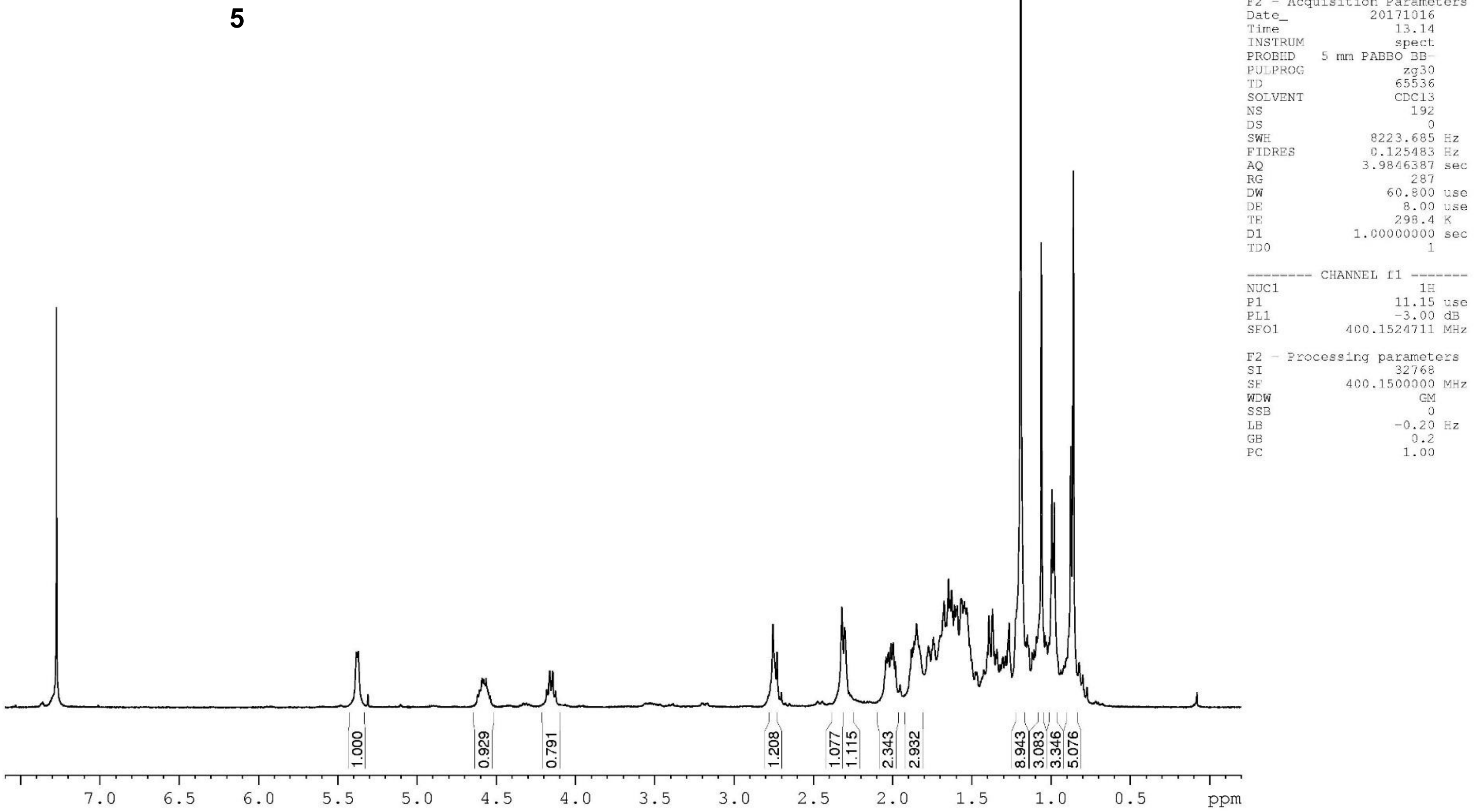
Current Data Parameters
NAME AW G282''(3)oczysz
EXPNO 1
PROCNO 1
F2 - Acquisition Parameters
Date_ 20171016
Time 13.14
INSTRUM spect
PROBHD 5 mm PABBO BB-
PULPROG zg30
TD 65536
SOLVENT CDCl3
NS 192
DS 0
SWH 8223.685 Hz
FIDRES 0.125483 Hz
AQ 3.9846387 sec
RG 287
DW 60.800 use
DE 8.00 use
TE 298.4 K
D1 1.00000000 sec
TD0 1
======== CHANNEL f1 ========
NUC1 1H
P1 11.15 use
PL1 -3.00 dB
SFO1 400.1524711 MHz
F2 - Processing parameters
SI 32768
SF 400.1500000 MHz
WDW GM
SSB 0
LB -0.20 Hz
GB 0.2
PC 1.00
1.000
0.929
0.791
1.208
1.077
1.115
2.343
2.932
8.943
3.083
3.346
5.076
7.0
6.5
6.0
5.5
5.0
4.5
4.0
3.5
3.0
2.5
2.0
1.5
1.0
0.5
ppm

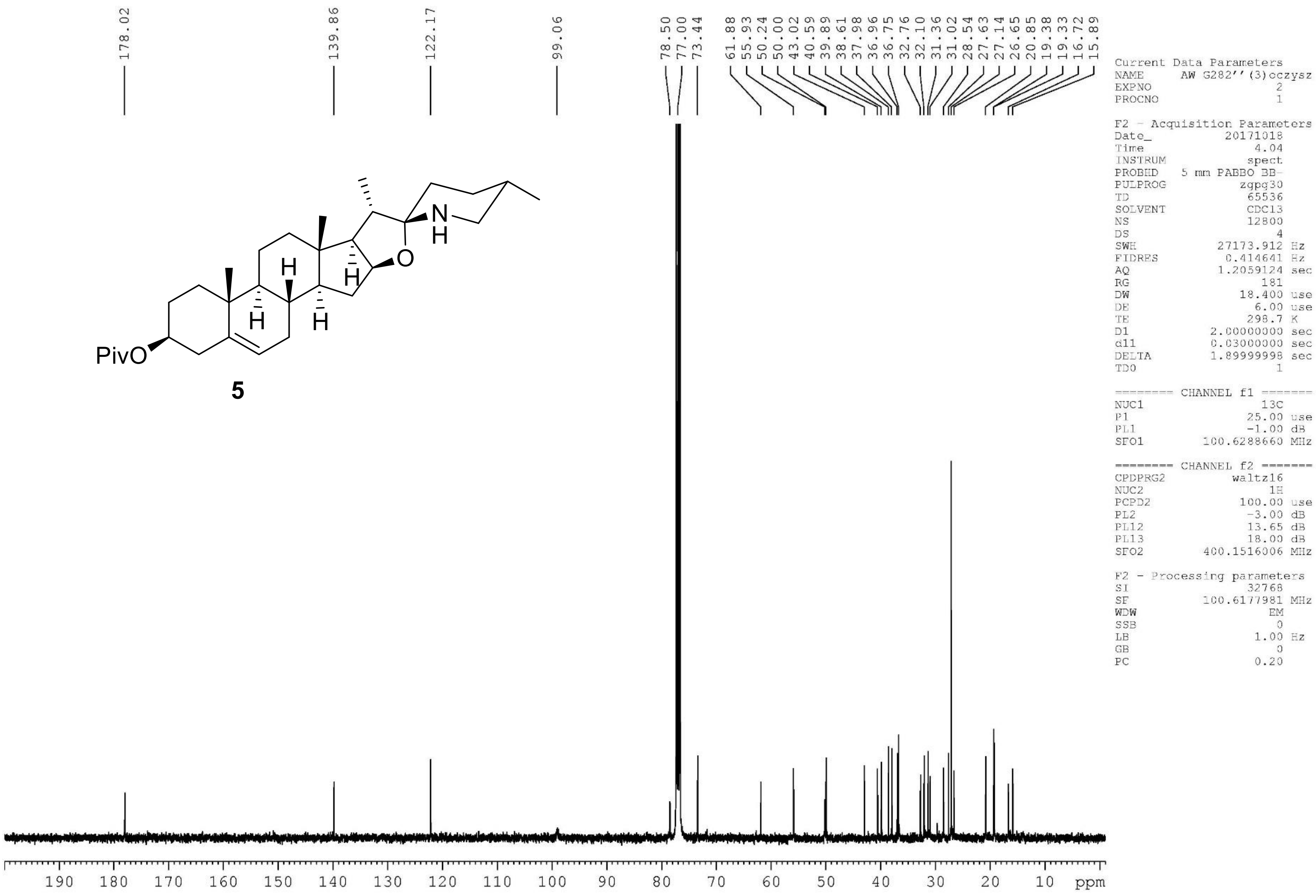

178.02
139.86
122.17
99.06
78.50
77.00
73.44
61.88
55.93
50.24
50.00
43.02
40.59
39.89
38.61
37.98
36.96
36.75
32.76
32.10
31.36
31.02
28.54
27.63
27.14
26.65
20.85
19.38
19.33
16.72
15.89
PivO
N
H
H
H
H
H
O
5
Current Data Parameters
NAME AW G282''(3)oczysz
EXPNO 2
PROCNO 1
F2 - Acquisition Parameters
Dato_ 20171018
Time 4.04
INSTRUM spect
PROBHD 5 mm PABBO BB-
PULPROG zgpg30
TD 65536
SOLVENT CDC13
NS 12800
DS 4
SWH 27173.912 Hz
FIDRES 0.414641 Hz
AQ 1.2059124 sec
RG 181
DW 18.400 use
DE 6.00 use
TE 298.7 K
D1 2.00000000 sec
d11 0.03000000 sec
DELTA 1.89999998 sec
TD0 1
======== CHANNEL f1 ========
NUC1 13C
P1 25.00 use
PL1 -1.00 dB
SFO1 100.6288660 MHz
======== CHANNEL f2 ========
CPDPRG2 waltz16
NUC2 1H
PCPD2 100.00 use
PL2 -3.00 dB
PL12 13.65 dB
PL13 18.00 dB
SFO2 400.1516006 MHz
F2 - Processing parameters
SI 32768
SF 100.6177981 MHz
WDW EM
SSB 0
LB 1.00 Hz
GB 0
PC 0.20
190 180 170 160 150 140 130 120 110 100 90 80 70 60 50 40 30 20 10 ppm

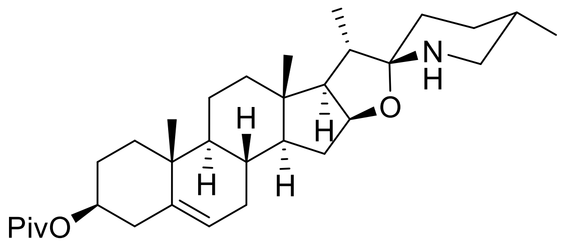

PivO
N
H
O
H
H
H
H

5

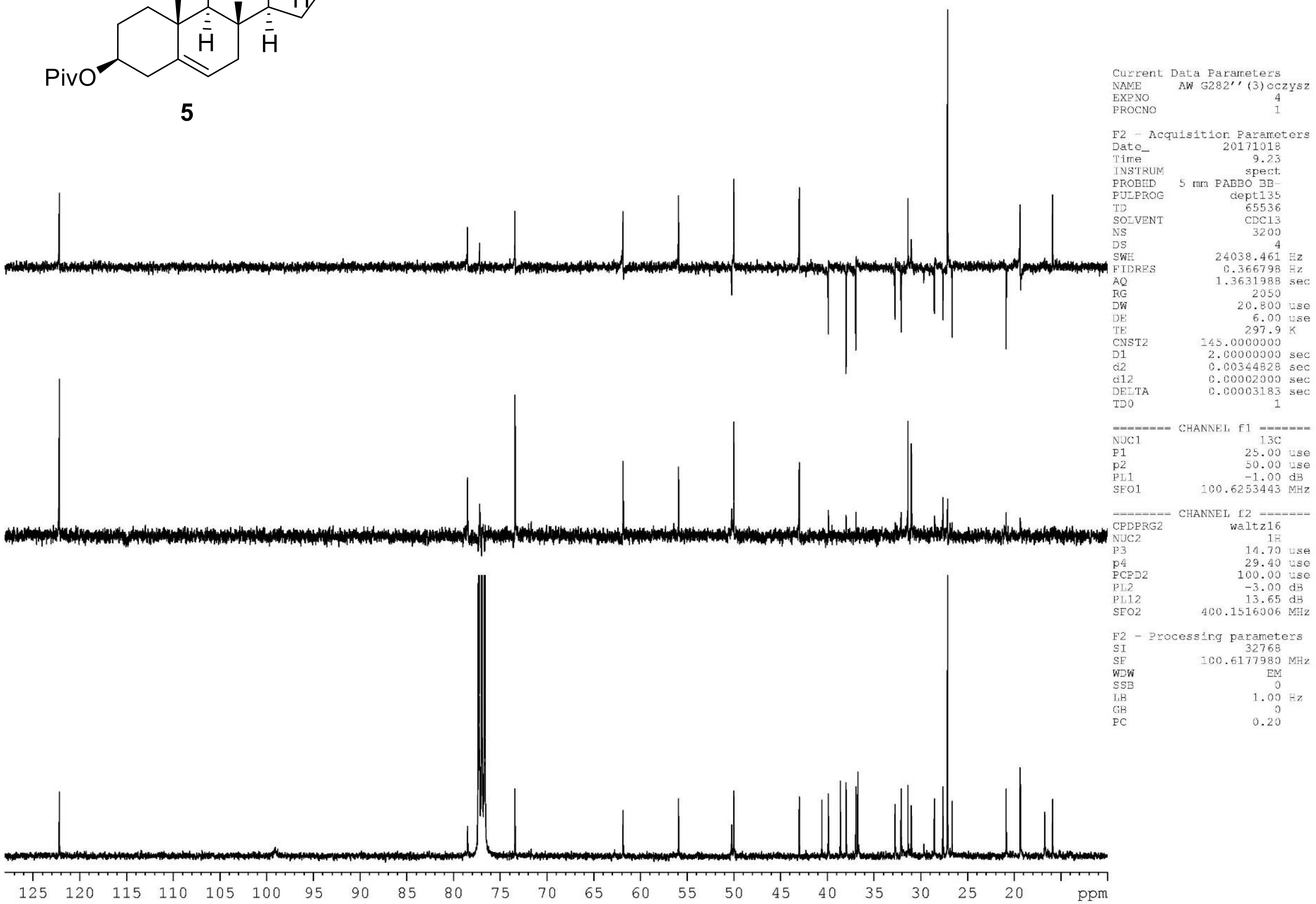

Current Data Parameters
NAME AW G282''(3)oczysz
EXPNO 4
PROCNO 1
F2 - Acquisition Parameters
Dato_ 20171018
Time 9.23
INSTRUM spect
PROBHD 5 mm PABBO BB-
PULPROG dept135
TD 65536
SOLVENT CDCl3
NS 3200
DS 4
SWH 24038.461 Hz
FIDRES 0.366798 Hz
AQ 1.3631988 sec
RG 2050
DW 20.800 use
DE 6.00 use
TE 297.9 K
CNST2 145.0000000
D1 2.00000000 sec
d2 0.00344828 sec
d12 0.00002000 sec
DELTA 0.00003183 sec
TD0 1
======== CHANNEL f1 =======
NUC1 13C
P1 25.00 use
p2 50.00 use
PL1 -1.00 dB
SFO1 100.6253443 MHz
======== CHANNEL f2 =======
CPDPRG2 waltz16
NUC2 1H
P3 14.70 use
p4 29.40 use
PCPD2 100.00 use
PL2 -3.00 dB
PL12 13.65 dB
SFO2 400.1516006 MHz
F2 - Processing parameters
SI 32768
SF 100.6177980 MHz
WDW EM
SSB 0
LB 1.00 Hz
GB 0
PC 0.20
125 120 115 110 105 100 95 90 85 80 75 70 65 60 55 50 45 40 35 30 25 20 ppm
